# HSV-1 triggers paracrine fibroblast growth factor response from cortical brain cells via immediate-early protein ICP0

**DOI:** 10.1186/s12974-019-1647-5

**Published:** 2019-12-02

**Authors:** Niko Hensel, Verena Raker, Benjamin Förthmann, Nora Tula Detering, Sabrina Kubinski, Anna Buch, Georgios Katzilieris-Petras, Julia Spanier, Viktoria Gudi, Sylvia Wagenknecht, Verena Kopfnagel, Thomas Andreas Werfel, Martin Stangel, Andreas Beineke, Ulrich Kalinke, Søren Riis Paludan, Beate Sodeik, Peter Claus

**Affiliations:** 1 Institute of Neuroanatomy and Cell Biology, Hannover Medical School, Hannover, Germany; 2 Niedersachsen-Research Network on Neuroinfectiology (N-RENNT), Hannover, Germany; 3 Center for Systems Neuroscience (ZSN), Hannover, Germany; 4 Institute of Virology, Hannover Medical School, Hannover, Germany; 5 German Center for Infection Research (DZIF), Hannover-Braunschweig, Germany; 6 Department of Biomedicine, Aarhus University, Aarhus, Denmark; 7 Institute for Experimental Infection Research, TWINCORE, Centre for Experimental and Clinical Infection Research, a joint venture between the Hannover Medical School and the Helmholtz Centre for Infection Research, Hannover, Germany; 8 Clinical Neuroimmunology and Neurochemistry, Department of Neurology, Hannover Medical School, Hannover, Germany; 9 Division of Immunodermatology and Allergy Research, Department of Dermatology and Allergy, Hannover Medical School, Hanover, Germany; 10 Department of Pathology, University of Veterinary Medicine Hannover, Hannover, Germany; 11 Sahlgrenska Academy, University of Gothenburg, Gothenburg, Sweden

**Keywords:** Neurotrophic factors, Fibroblast growth factors, FGF, ERK, Akt, Signaling, HSV-1, ICP0, Cortex

## Abstract

**Background:**

Herpes simplex virus-1 (HSV-1) infections of the central nervous system (CNS) can result in HSV-1 encephalitis (HSE) which is characterized by severe brain damage and long-term disabilities. Different cell types including neurons and astrocytes become infected in the course of an HSE which leads to an activation of glial cells. Activated glial cells change their neurotrophic factor profile and modulate inflammation and repair. The superfamily of fibroblast growth factors (FGFs) is one of the largest family of neurotrophic factors comprising 22 ligands. FGFs induce pro-survival signaling in neurons and an anti-inflammatory answer in glial cells thereby providing a coordinated tissue response which favors repair over inflammation. Here, we hypothesize that FGF expression is altered in HSV-1-infected CNS cells.

**Method:**

We employed primary murine cortical cultures comprising a mixed cell population of astrocytes, neurons, microglia, and oligodendrocytes. Astrocyte reactivity was morphometrically monitored by an automated image analysis algorithm as well as by analyses of A1/A2 marker expression. Altered FGF expression was detected by quantitative real-time PCR and its paracrine FGF activity. In addition, HSV-1 mutants were employed to characterize viral factors important for FGF responses of infected host cells.

**Results:**

Astrocytes in HSV-1-infected cortical cultures were transiently activated and became hypertrophic and expressed both A1- and A2-markers. Consistently, a number of FGFs were transiently upregulated inducing paracrine neurotrophic signaling in neighboring cells. Most prominently, FGF-4, FGF-8, FGF-9, and FGF-15 became upregulated in a switch-on like mechanism. This effect was specific for CNS cells and for a fully functional HSV-1. Moreover, the viral protein ICP0 critically mediated the FGF switch-on mechanism.

**Conclusions:**

HSV-1 uses the viral protein ICP0 for the induction of FGF-expression in CNS cells. Thus, we propose that HSV-1 triggers FGF activity in the CNS for a modulation of tissue response upon infection.

## Background

Herpes simplex encephalitis (HSE) may cause severe brain damage and is one of the most common causes for infectious encephalitis [1, 2]. Lethality dramatically decreased with the introduction of antiviral acyclovir therapy, however, between 5 and 20% of the patients decease during the course of an HSE [2]. Moreover, a high percentage of survivors suffer from severe long-term disabilities such as memory deficits, personality and behavioral changes, as well as psychiatric disorders [3]. Only a few HSE cases are associated with HSV-2. The vast majority is caused by HSV-1 infections [4, 5]. The double-stranded DNA-virus occasionally causes a neuroinflammation in cortical regions such as the medial temporal lobe which could account for memory deficits in surviving patients [6]. The seroprevalence of HSV-1 ranges from 60 to 90% in adults [7]. Considering this, the HSE incidence of 1 in 250,000 to 1 in 500,000 is a rare event [8].

In most cases, HSV-1 infections are not critical. An initial primary infection of epithelial cells allows the virus to enter free nerve endings of sensory neurons followed by a retrograde transport to cell bodies in the trigeminal ganglion [9]. Here, HSV-1 establishes life-long latency characterized by an expression of only a few viral proteins [10–12]. Stress-associated immunosuppression reactivates the virus leading to an anterograde transport of newly built viral particles and in most cases to the occurrence of herpes labialis, also known as cold sores [10, 13]. About one-third of the HSE cases are caused by primary infections most commonly in younger patients, whereas two-thirds of the cases were HSV-1 seropositive before disease onset [4, 8]. Immunosuppressed HSE patients have an enhanced mortality compared to immunocompetent subjects [14]. However, they do not exhibit an enhanced susceptibility to HSV-1 CNS infections [15]. Thus, immunity critically influences the outcome of an HSE, but other mechanisms may contribute to viral spread and damage within the CNS. The induction of neurotrophic repair-mechanisms ameliorates the symptoms of another infectious encephalitis caused by human immunodeficiency virus (HIV) [16]. This supports the hypothesis that neurotrophic signaling may also play a role in HSE.

In ischemic brain damage, an acute inflammatory phase is followed by an anti-inflammatory repair phase [17]. Inflammation includes the removal of damaged cells via apoptosis while the repair-phase is characterized by an anti-apoptotic and pro-survival environment [18]. These at least partially exclusive functions are coordinated by an extensive crosstalk between neurotrophic and inflammatory signaling [19]. Moreover, the same cell types execute both functions and therefore undergo substantial morphological and secretory changes. Microglia, the resident macrophages of the CNS, show multiple roles with inflammatory cells denoted M1- and repair cells M2-microglia. M1-microglia secrete TNF-α which in turn activates astrocytes in a pro-inflammatory mode [20]. Those pro-inflammatory A1 astrocytes secrete chemokines such as CXCL10 before they become anti-inflammatory A2 astrocytes during the repair phase. A2 astrocytes express markers such as Cox2 [21] and provide a repair-environment by secretion of neurotrophic factors [22, 23]. Neurotrophic factors include the neurotrophin family with nerve growth factor (NGF), brain-derived neurotrophic factor (BDNF), and neurotrophins (NT). Other neurotrophic factors are ciliary neurotrophic factor (CNTF), glial-derived neurotrophic factor (GDNF), and the fibroblast growth factor family (FGF) [24]. The FGF-family comprises 22 ligands and four cognate FGF-receptors (FGFR) which are expressed in neurons, astrocytes, and microglia [25, 26]. The classical neurotrophic FGF-signaling results in a shift from an apoptotic to a regenerative pro-survival answer in neurons while FGFR activity in astrocytes and microglia leads to an anti-inflammatory response [27–29]. Thus, FGF-ligand secretion is an important mechanism for the coordinated brain tissue response during inflammatory conditions.

Here, we hypothesize that HSV-1 infection of CNS cells changes their activation state as well as their FGF-expression. We took advantage of a primary murine cortical culture comprising different CNS cell types including microglia, astrocytes, oligodendrocytes, and neurons. HSV-1 infection led to a transient astrocyte hypertrophy which was accompanied by expression of astrocyte activation markers. Interestingly, the CNS-cell culture responded with an increased expression of several FGF ligands in a switch-on like mechanism resulting in enhanced paracrine FGF activity. The FGF response was restricted to CNS cells and specifically caused by the HSV-1 virus. The use of viral knockout strains revealed a critical role of the viral protein ICP0 for HSV-1 triggered FGF expression.

## Methods

### Animals and viruses

All animals were handled in compliance with the regulations of the German animal welfare law. C57BL/6JHanZtm mice were obtained from the Central Animal Facility of Hannover Medical School, Germany. STING(-/-) [30] and MyD88(-/-) (Myd88^tm1Aki^) [31] knockout mice were kept at TWINCORE, Centre for Experimental and Clinical Infection Research, Hannover, Germany. The following virus strains have been used: HSV1(17^+^)Lox [32], HSV1(17^+^)Loxluc Δγ34.5 [32], HSV-1(F) (ATCC VR733), HSV-1-ΔUS11 [33], and HSV1(17^+^)Lox-_pMCMV_GFP [34]. Briefly, virus particles were harvested from the medium of infected BHK cells (baby hamster kidney cells) by sedimentation and titrated on Vero cells as described before [34, 35]. Additionally, we employed HSV1 KOS ΔICP0 (7134) [36], HSV1 KOS1.1 ICP22 (d22) [37], HSV1 KOS ICP4 (vi13) [38], and HSV1 KOS ICP27 (d27.1) [39] strains as well as Theiler’s murine encephalomyelitis virus (TMEV; strain BeAn) [40].

### Preparation of primary cortical cells

Whole cortices of neonatal mice (P1–P6) were dissected and dissociated employing an enzymatic solution containing papain (25 U/ml in DMEM-GlutaMAX™ (Gibco), 1.65 mM L-cysteine (Sigma-Aldrich), 1 mM CaCl_2_, 0.5 mM EDTA). After 20 min at 37 °C, the enzymatic solution was replaced by an inactivating solution (DMEM-GlutaMAX™, 2.5 mg/ml BSA (Sigma-Aldrich), 2.5 mg/ml trypsin inhibitor (Sigma-Aldrich), 10% fetal bovine serum (FBS, PAA Laboratories), 100 U/ml penicillin/streptomycin (Invitrogen), 1× MITO+ (BD Biosciences)), and incubated for 5 min at room temperature. The supernatant was discarded and the tissue was sheared in FBS-medium (DMEM-GlutaMAX™, 10% FBS, 100 U/ml penicillin/streptomycin, 1× MITO+). After tissue parts settled down, the supernatant was centrifuged (5 min, 157×*g*) and the cell pellet was resuspended in FBS-medium. The cells were seeded on poly-l-lysine (PLL)–coated (0.5 ng/ml, Sigma-Aldrich) well plates in FBS-medium. Thirty minutes after incubation (37 °C, 5% CO_2_), the FBS-medium was replaced by NBA-medium (NeurobasalA® (Gibco), B27 (Invitrogen), GlutaMAX™ (Invitrogen), 100 U/ml penicillin/streptomycin). Half of the NBA-medium was exchanged with fresh medium 24 h later (day in vitro, DIV2).

### Primary astrocytes and C127i cell line

Murine primary astrocytes derived from C57BL/6JHanZtm neonatal mice were prepared as described previously [41] with a purity of about 90% [42]. Astrocytes were cultured in DMEM-high glucose (Gibco) supplemented with 10% fetal bovine serum (FBS, PAA Laboratories) and 100 U/ml penicillin/streptomycin (Invitrogen). Murine mammary gland epithelial cells (C127i, ATCC CRL-1616) were cultured in DMEM (4.5 g/L glucose; Gibco) supplemented with 10% FBS and 100 U/ml penicillin/streptomycin and incubated at 37 °C and 5% CO_2_.

### Primary human keratinocytes

NHEK-Neo keratinocytes (Neonatal Normal Human Epidermal Keratinocytes) from Lonza (Basel, Switzerland) were used. Keratinocytes were passaged and cultured in Keratinocyte Growth Medium 2 kit (PromoCell). At a confluency of 70–80%, cells were employed for further experiments or passaged. Cells were used between passage 8 and 10.

### Infection and treatment of cell cultures

Primary cortical cells (DIV5), primary astrocytes, or C127i cells were incubated with CO_2_-independent medium (Gibco) containing 0.1% (w/v) BSA (1 ml/6-well; 0.2 ml/24-well) for 20 min at room temperature on a rocking platform. Viral particles were suspended in fresh CO_2_-independent medium containing 0.1% (w/v) BSA and incubated with the cells in a multiplicity of infection of 10 plaque forming units per cell (MOI 10). During infection, the cells were placed on a rocking platform for 30 min at room temperature. After exchange of infectious medium to culture medium (NeurobasalA® (Gibco), GlutaMAX™ (Invitrogen), 100 U/ml penicillin/streptomycin, B27) the cells were incubated at 37 °C for different time points. For stress induction in similar conditions to infected cells, cultures were incubated with CO_2_-independent medium containing 0.1% (w/v) BSA for 50 min. Subsequently, primary cortical cells were incubated with dithiothreitol (1 mM) in NBA-medium for 6 h. Toll-like receptor (TLR) agonists of the mouse TLR1-9 Agonist Kit (Invitrogen) were used according to manufacturer’s instruction, diluted in NBA-medium, and incubated on cells for 6 h at 37 °C and 5% CO_2_ (Pam3CSK4, LPS-EK, FLA-ST, FSL-1: 100 μg/ml; HKLM: 10^10^ cells/ml; Poly(I:C) (HMW) and (LMW): 1 mg/ml; ssRNA40: 50 μg/ml; ODN1826: 500 μM). Cells were washed with PBS once and used for RNA isolation. Keratinocytes were infected with a multiplicity of infection (MOI) of 10 and incubated for 1 h at 37 °C and 5% CO_2_ atmosphere in 170 μL CO_2_-independent medium (Gibco). Afterwards, the CO_2_-independent medium was replaced by 400 μL Keratinocyte Growth Medium 2. After 6 h at 37 °C and 5% CO_2_ atmosphere, the cells were washed with PBS once and directly lyzed for RNA isolation.

### UV inactivation of HSV-1

HSV-1(17^+^)Lox was diluted in CO_2_-independent medium with 0.1% BSA and inactivated using an UV transilluminator (FLX-20.M; Vilber Lourmat, France) with different UV dosages (0.1–0.8 J/cm^2^). Control medium and non-inactivated control were treated the same omitting the UV radiation.

### Induction of cells with conditioned medium

After infection of primary cortical cultures, the infectious medium was changed to starvation medium (culture medium without B27) and incubated for different time points. The supernatant was filtered with a syringe filter (Millex-VV, 0.1 μm, PVDF; Merck Millipore) to clear the conditioned medium of viral particles. Plaque assays confirmed the absence of viral particles [43]. Briefly, just-confluent Vero cells were incubated with a dilution series of unfiltered and filtered medium for 1 h at room temperature. The inoculum was changed to growth medium containing 10 μg/ml human IgG (Sigma-Aldrich). After 2 days of culture, cells were fixed and stained with 0.1% crystal violet in 2% ethanol for plaque visualization. The conditioned media were incubated together with non-infected primary cortical cultures which were starved with starvation medium 2 h prior to this induction. After 2 h of induction, cells were lyzed for Western blot analysis or real-time PCR. For inhibition of the fibroblast growth factor receptor (FGFR), the inhibitor PD173074 (Calbiochem) was added to starvation medium or conditioned media to a final concentration of 200 nM, respectively. To efficiently inhibit the FGFR-receptors, PCCs were pre-treated with the inhibitor 2 h before addition of conditioned medium. Control cells received starvation medium or conditioned medium with same concentration of the inhibitor-vehicle DMSO.

### RNA isolation, reverse transcription, and real-time PCR

RNA was isolated with RNeasy® Plus Mini Kit (Qiagen) according to the manufacturer’s guidelines. RNA Integrity Numbers (RIN) were determined with a Bioanalyzer using the RNA Nano Kit 6000 (Agilent Technologies). Samples with RIN < 8 were omitted from further analysis. 0.5–1 μg of total RNA was pre-incubated with 3 μg Random Primers (Invitrogen) at 70 °C for 2 min followed by a cooling step on ice. Reverse transcription was performed in First Strand Buffer (Invitrogen) containing 10 mM DTT (Invitrogen), 0.5 mM dNTPs (Invitrogen), 5 U/ml M-MLV Reverse Transcriptase (Invitrogen) and 1 U/ml RNase Block Ribonuclease Inhibitor (Agilent Technologies). The reaction mix was incubated for 90 min at 42 °C, 15 min at 70 °C and cooled down on ice. In case of low RNA-yields, we used iScript^TM^ cDNA Synthesis Kit (Bio-Rad) according to the manufacture’s guidelines. For real-time PCR, the cDNA was diluted 1:25. Five microliters of diluted cDNA was mixed with 7 μl Power SYBR® Green Master Mix (Applied Biosystems) and 2 μl diluted primer mix (1.75 μM each forward and reverse primer). PCR reaction was performed with a StepOnePlus™ real-time PCR system. The temperature protocol included a first denaturation step of 10 min at 95 °C followed by 40 cycles with 15 s at 95 °C and 1 min at 60 °C. PCR product specificity was verified by melting curve analysis. FGF primers have been used and validated previously [44], novel primer sequences are shown in Additional file 1: Table S1.

### Conventional PCR and gel electrophoresis

cDNA and RNA derived from HSV-1 infected and control primary cortical cells were amplified using FGF-4 primers covering both introns (Additional file 1: Figure S2), respectively. Thermocycler performed an initial denaturation step at 95 °C (3 min) followed by 40 cycles of 95 °C (30 s), 62.4 °C (30 s), 72 °C (30 s), and a final extension (72 °C, 5 min).

### Western blot

Cells were lyzed with RIPA buffer (137 mM NaCl, 20 mM Tris-HCl pH 7, 525 mM β-glycerophosphate, 2 mM EDTA, 1 mM sodium-orthovanadate, 1% (w/v) sodium-desoxycholate, 1% (v/v) Triton-X-100, protease inhibitor cocktail (Roche). Lysates were stored on ice for 15 min, sonicated for 15 min, and centrifuged at 4 °C for 20 min (22,000 rcf). Protein concentration of the supernatant was analyzed with Pierce™ BCA Protein Assay kit. The following antibodies were used on Western blots after SDS-PAGE: primary antibodies; Akt (pan) (1:1000; Cell Signaling), Phospho-Akt (Ser473) (1:1000; Cell Signaling); p44/42 MAPK (Erk1/2) (1:1000; Cell Signaling), Phospho-p44/42 MAPK (Erk1/2) (Thr202/Tyr204) (197G2) (1:1000; Cell Signaling), Tubulin (DM1A) (1:3000; Santa Cruz), self-raised polyclonal rabbit anticapsid antibody HSV-1 [45]. HRP-linked secondary antibodies: anti-mouse IgG (1:4000; GE Healthcare), anti-rabbit IgG (1:5000; GE Healthcare). Primary antibodies directed against non-phospho epitopes, as well as secondary antibodies, were diluted in 5% milk powder in TBS-T, primary phospho-antibodies in 5% BSA in TBS-T. Detection of chemiluminescence was performed with Immobilon™ Western HRP Substrate (Millipore).

### Immunocytochemistry

Cells were grown on PLL-coated glass cover slips in 24-well plates. After treatment or infection, cells were washed with PBS once and fixed with 4% (w/v) paraformaldehyde (Sigma-Aldrich) in PBS for 10 min at room temperature following permeabilization with ice cold methanol at – 20 °C for 10 min. Cells were washed with PBS and permeabilized with 0.3% Triton X-100 in PBS containing 3% normal goat serum (Gibco), 1% bovine serum albumin (Sigma-Aldrich) and 5% human serum from HSV-1-seronegative donors [43]. Primary antibodies (βIII-tubulin, 1:500, Millipore; GFAP, mouse, 1:500, Sigma-Aldrich; Iba-1, 1:500, Wako Chemicals; Olig2, 1:500, Millipore; FGF-9, 1:100, Santa Cruz) were diluted in blocking solution with 0.3% Triton X-100 and incubated on cells overnight at 4 °C. Cells were incubated with fluorescent secondary antibodies (anti-mouse-AlexaFluor555, goat, 1:500; Molecular Probes) for 1 h at room temperature. DAPI (1:2000; Sigma-Aldrich) staining of nuclei was performed during PBS washing steps. After staining, cover slips were mounted on object slides with ProLong® Gold Antifade Mountant (Molecular Probes). Microscopy was performed with an Olympus BX61 epifluorescence microscope.

### Automated image analysis

Microscope images of astrocytes were analyzed using the open-source cell image analysis software CellProfiler 2.2.0 [46]. The pipeline for astrocyte analysis is provided in the supplement.

### Statistics

Data analysis was performed with GraphPad Prism 6.07 (GraphPad Software, Inc., La Jolla, USA). Grouped data were analyzed with repeated-measures one-way or two-way ANOVA followed by Holm-Šídák multiple comparison corrected post-test. Statistical evaluation of gene expression data was performed with transcript levels (tl) which were calculated relative to the housekeeping 18S: tl = 2^ΔCt^ with *ΔCt = Ct*_*18S*_ *− Ct*_*target-of-interest*_. Fold changes were calculated using the ΔΔCt-method. Fold changes from samples with no expression were calculated setting the Ct-value to 40. Densitometric data was normalized to the geometric mean of all values from the same blot and the statistical analysis was performed with relative phospho to non-phospho ratios.

## Results

### HSV-1 preferentially infects astrocytes which become activated in a mixed cortical culture

**Fig. 1:**
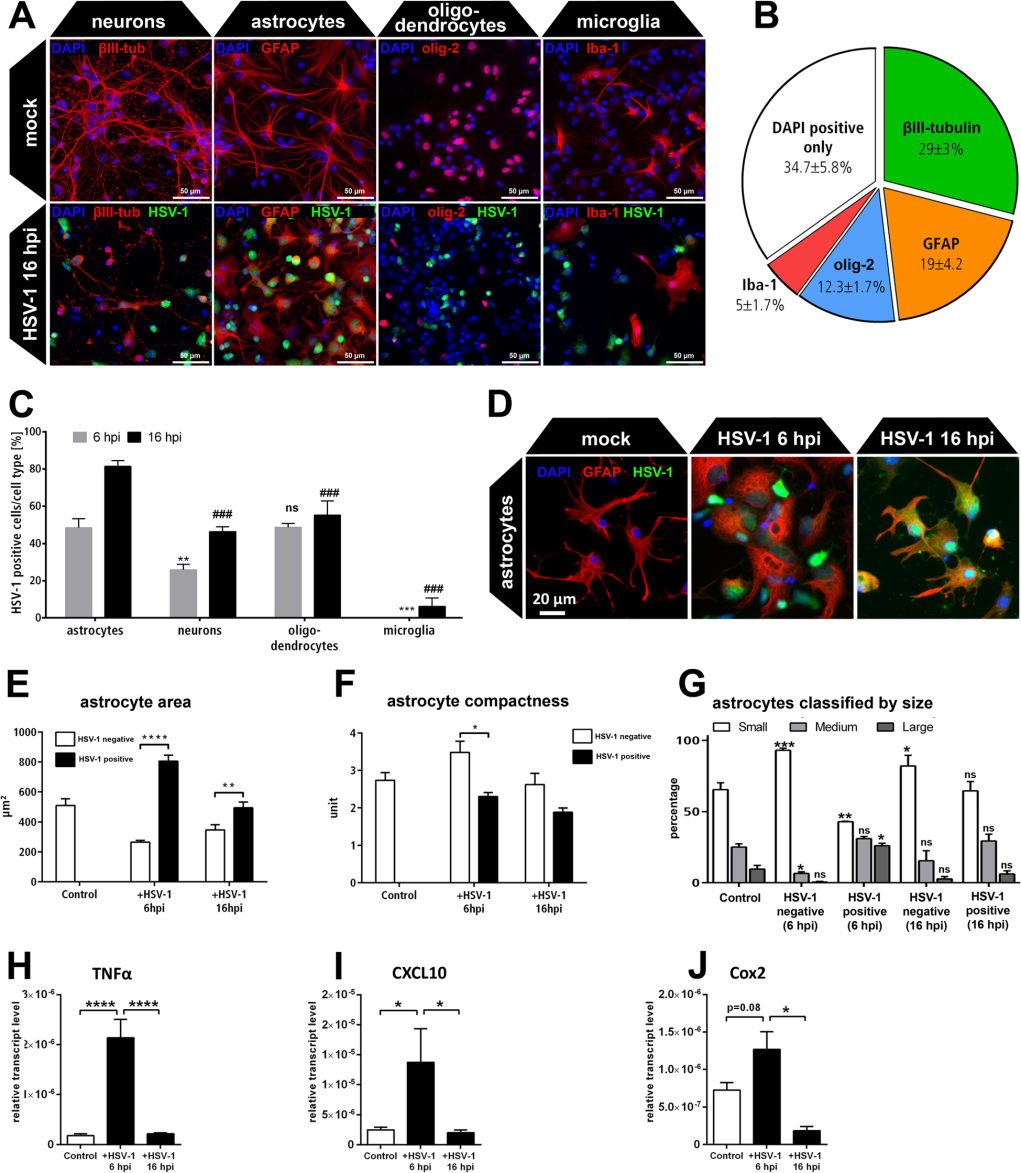
Characterization of HSV-1-infected primary cortical neurons (PCC). **a** Murine PCCs were infected with HSV-1(17^+^)Lox_pCMV_GFP (MOI 10) at DIV5 and compared with mock-infected control cells 16 hpi. Cells were stained against the neuronal marker βIII-tubulin (βIII-tub), the astrocytic marker glial fibrillary acidic protein (GFAP), the oligodendrocyte transcription factor (olig-2), and allograft inflammatory factor (Aif1/Iba-1) as a marker for microglia. **b** Cell type composition of the mock infected PCCs. **c** Percentage of HSV-1 positive cells 6 and 16 hpi defined for each cell type. Bars show mean ± SEM (*n* = 3) with a two-way ANOVA and a Holm-Sidak’s multiple comparison test (**p < 0.01, ***p < 0.001 compared to 6 hpi astrocytes, ^###^*p* < 0.001 compared to 16 hpi astrocytes). **d** The astrocytes in the PCCs were HSV-1(17^+^)Lox_pCMV_GFP infected (MOI 10) and analyzed 6 hpi and 16 hpi via GFAP staining. **e**–**g** GFAP positive astrocytes were characterized using the automated cell image analysis software CellProfiler. **e** The area of HSV-1 negative and HSV-1-positive astrocytes was measured within mock control and HSV-1-infected PCCs. **f** Compactness of infected and non-infected astrocytes. **g** Classification of HSV-1 positive and HSV-1 negative astrocytes depending on the area of the cell body related to the total astrocyte area (large > 1000 μm^2^, medium 1000 μm^2^ ≥ × ≤ 500 μm^2^, small < 500 μm^2^). Sidak’s multiple comparison tests refer to mock-infected control astrocytes of the same size-class. **h**–**j** mRNA levels of A1/A2 markers were quantified by qRT-PCR in PCCs 6 and 16 hpi. All bars show mean ± SEM (*n* = 3) with a two-way ANOVA (**e**–**g**) and a one-way ANOVA (**h**–**j**) followed by Sidak’s multiple comparison test (****p < 0.0001, **p < 0.01, *p < 0.05)

Here, we investigated the neurotrophic FGF-expression of HSV-1-infected CNS cells. The CNS comprises various cell types which co-regulate tissue response upon brain damage such as microglia modulating astrocyte responses [20], a major source of neurotrophic factors in the damaged brain [23]. Therefore, we employed primary murine cortical cell cultures (PCCs) comprising a mixed CNS cell population such as neurons and glial cells similar to the in vivo situation [47]. We dissected both cortical hemispheres including the temporal lobe—the main regions affected during HSV-1 encephalitis in humans [6]. The cell type composition of primary cortical cells was characterized by neuronal, astrocytic, oligodendrocytic, and microglial marker staining (Fig. 1a). Apart from undefined cells, the culture mainly comprises neurons, followed by astrocytes, oligodendrocytes, and microglia (Fig. 1b). These cultures were infected for 6 and 16 h with a genetically modified HSV-1(17^+^)Lox_pMCMV_GFP reporter strain expressing green fluorescent protein (GFP) (Fig. 1a). In this system, a higher proportion of astrocytes became infected by 6 hpi (48%). HSV-1-infected neurons represented a smaller fraction of only 26% at the same time point (Fig. 1c). The Iba-1 positive microglia did not show signs of HSV-1 infection except for a small fraction at 16 hpi (Fig. 1a, c). Thus, astrocytes were the second most abundant cell type in the culture and showed the highest infection rate compared to neurons and other glial cells. Moreover, they responded with an apparent morphological change (Fig. 1a).

We quantified the morphological changes of GFAP-positive astrocytes in PCCs 6 and 16 hpi using an automated and unbiased image analysis algorithm based on the software CellProfiler [46] (Fig. 1d). Thereby, we distinguished between infected astrocytes and non-infected neighboring astrocytes in the same culture (Fig. 1e–g). HSV-1 positive astrocytes became significantly larger compared to neighboring HSV-1 negative astrocytes at 6 hpi. After additional 10 h incubation, infected astrocytes reduced their size again and resembled the mock-infected control cells (Fig. 1e). Accordingly, the compactness of the astrocytes differed between HSV-1 negative and HSV-1 positive astrocytes after 6 hpi (Fig. 1f). The compactness describes the shape of cells and is calculated by the mean square distance of the cells border from the cell centroid divided by the area. A perfect circular cell would have a compactness of 1. As for infected astrocytes, a more compact shape was measured compared to HSV-1 negative and control cells. Indeed, control astrocytes displayed a ramified morphology compared to round-shaped infected cells (Fig. 1d).

The size distribution revealed a more detailed pattern of astrocyte activation in PCCs (Fig. 1g). In control conditions, over 60% of the astrocytes were small, 25% were categorized as medium and less than 10% of the cells were large. After 6 h of infection, HSV-1 negative and positive astrocytes changed their size distribution in opposite directions within the same culture: HSV-1 negative astrocytes became smaller with a reduced fraction of medium-sized and an enhanced fraction of small cells. HSV-1 positive astrocytes became larger indicated by an impressive reduction in the percentage of small astrocytes and an increase in large cells. At 16 hpi, there was an enhanced percentage of small astrocytes in HSV-1 negative cells while HSV-1 positive cells largely resembled the size-composition of mock-infected control cells (Fig. 1g). These results revealed a transient response of astrocytes to HSV-1 infection with two different cell populations: Non-infected cells became hypotrophic while infected astrocytes displayed a hypertrophic phenotype. Astrocytes can be activated to develop an inflammatory A1- or a neuroprotective A2-phenotype [20]. Thus, we measured the expression of A1/A2 markers in HSV-1-infected PCCs. Indeed, A1-markers TNFα and CXCL10 became transiently upregulated (Fig. 1g, h). Nevertheless, the A2 marker Cox2 was increased (Fig. 1i) which hints for a partially neurotrophic astrocytic response of HSV-1-infected PCCs.

### HSV-1 infection of primary cortical cells alters gene expression of the FGF-system

**Fig. 2:**
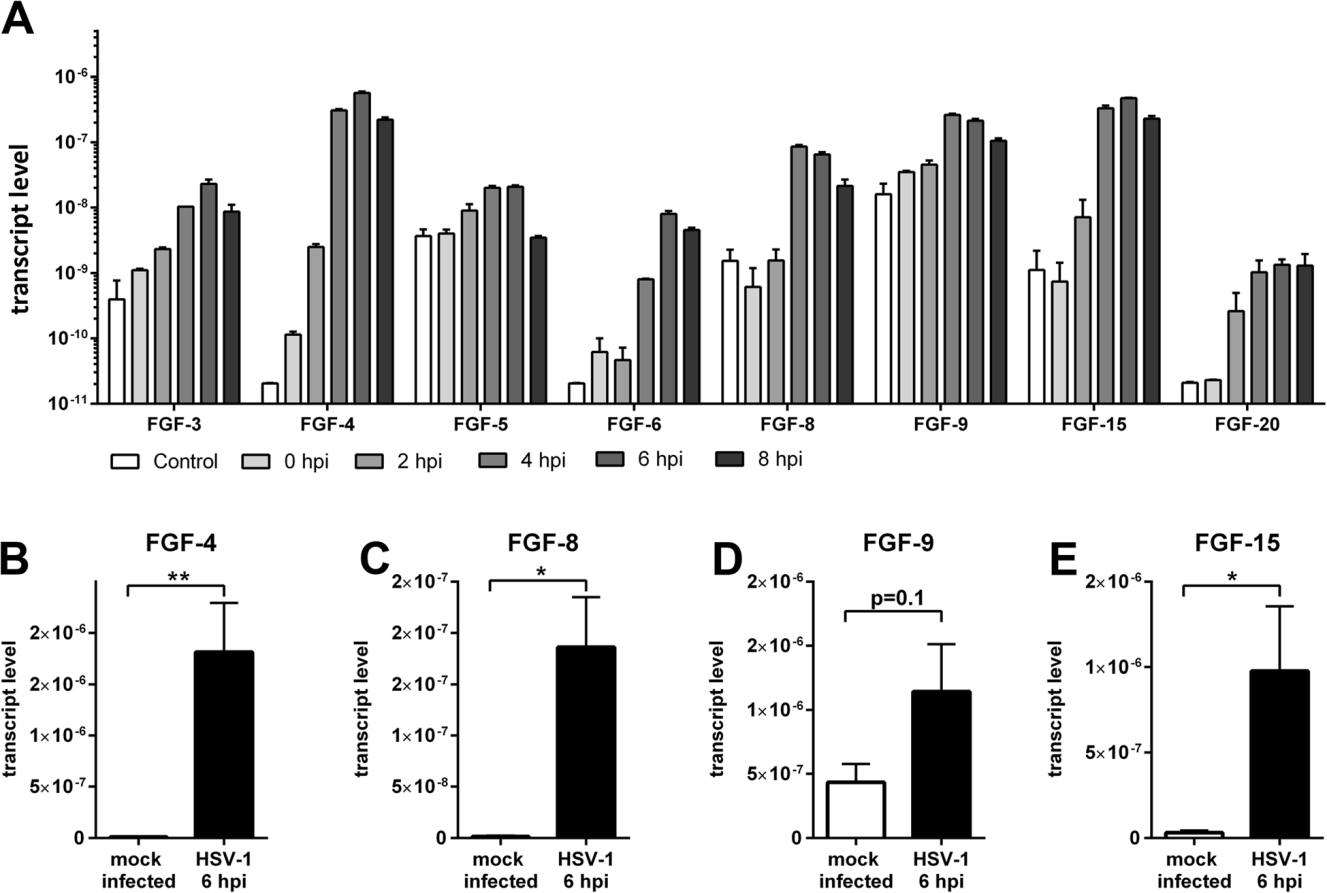
Upregulated fibroblast growth factors (FGFs) in response to HSV-1 infection. **a** FGF mRNAs were quantified by an initial qRT-PCR screening in control and HSV-1(17^+^)Lox-infected PCCs (MOI 10) 0, 2, 4, 6, or 8 hpi. 0 hpi cells were inoculated with HSV-1 for 30 min without any incubation in culture medium at 37 °C and 5% CO_2_. Instead, cells were immediately lyzed. **b**–**e** Validation of the screening results with an increased number of biological independent replicates. Bars show mean ± SEM with *n* = 2 for **a**, and *n* = 6 for **b**–**e**. Student’s *t* test with (**p < 0.01, *p < 0.05)

Astrocyte activation is associated with an altered secretory profile including neurotrophic factors [23] such as FGFs. Therefore, we screened the expression profile of the 22 members of the neurotrophic FGF-family by qRT-PCR. The transcription machinery in the host cell is strongly influenced by HSV-1 leading to a global mRNA downregulation [48]. Not surprisingly, several FGF transcripts were downregulated after HSV-1 infection (Additional file 1: Figure S1). However, the screening also identified positively regulated growth factors**.** The mRNA levels of FGF-3, 5, 6, and 20 were slightly enhanced on a low expressional level while FGF-4, 8, 9, and 15 were robustly upregulated after HSV-1 infection (Fig. 2a). Those FGFs were further validated with an increased number of replicates at 6 hpi (Fig. 2b–e). Indeed, FGFs-4, 8, and 15 were significantly upregulated. Interestingly, those ligands were almost not expressed in control cells indicating a switch-on mechanism in response to HSV-1 infection with FGF-4 being the most abundant mature mRNA transcript (Fig. 2 and Additional file 1: Figure S2). Moreover, FGF-9 was expressed in mock-infected control cells but displayed a tendency for an increase in response to HSV-1 infection.

### HSV-1 infected primary cortical cells secrete FGF ligands with paracrine activity

**Fig. 3:**
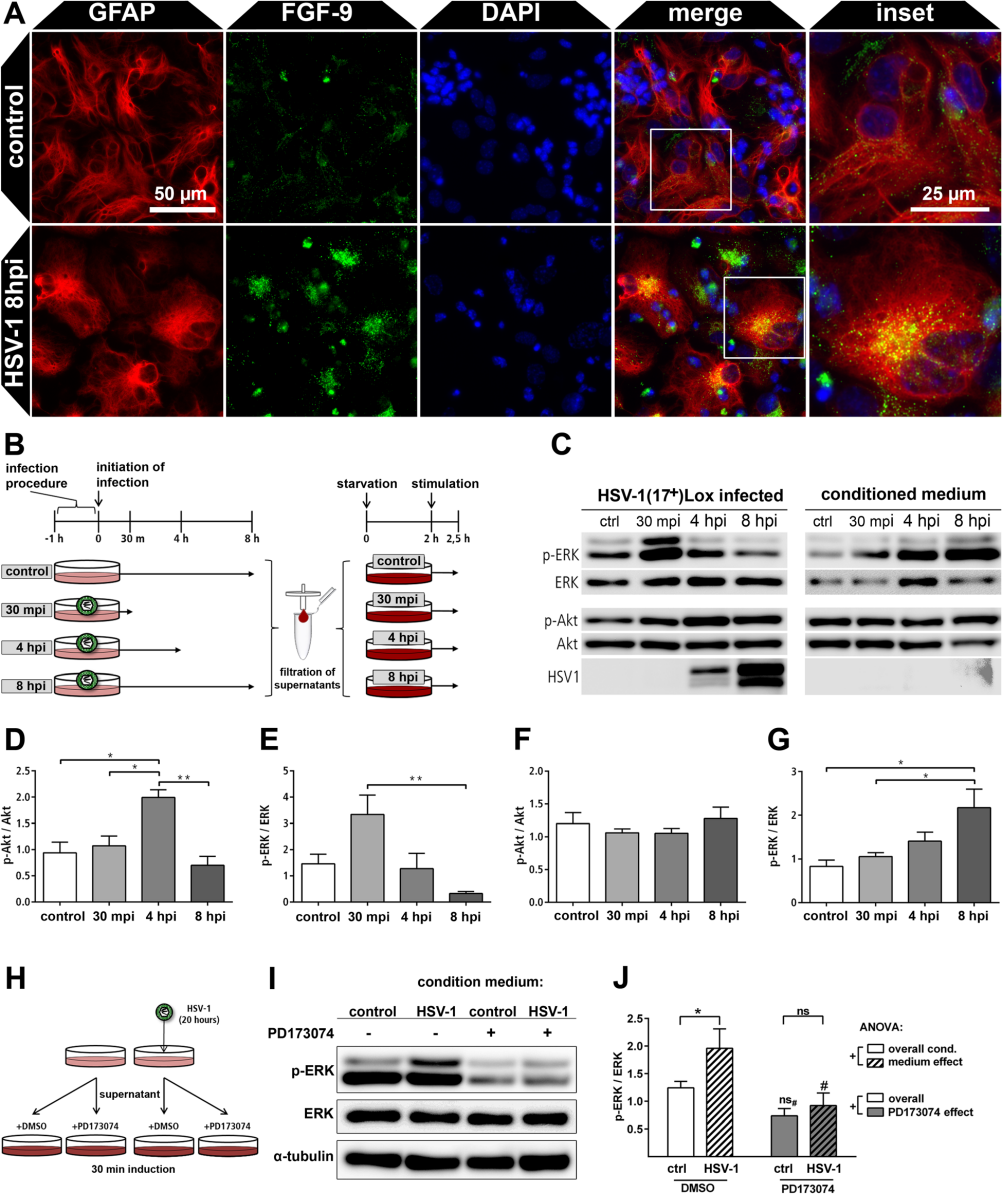
FGF-expression and paracrine induction of the ERK-pathway in response to HSV-1 infection of PCCs. **a** Mock-infected control PCCs and HSV-1(17^+^)Lox_pCMV_mCherry infected cells (8 hpi, MOI 10) were stained for GFAP and FGF-9. **b** HSV-1(17^+^)Lox infected PCCs were infected and the conditioned medium was collected 30 mpi, 4 hpi, and 8 hpi, while the supernatant media of control cells collected 8 h post mock-infection. Viral particles were removed by filtration. Non-infected PCCs were starved 2 h prior to conditioned medium incubation reducing pathway activities to a basal level. Subsequently, starved PCCs were incubated for 30 min with conditioned medium and immediately lyzed for Western blot analysis. **c** Representative Western blots of infected source cells and non-infected target cells treated with conditioned medium. **d**, **e** Densitometric analysis of ERK and Akt phosphorylation in HSV-1-infected source PCCs. **f**, **g** Densitometric analysis of ERK (**e**) and Akt (**f**) phosphorylation in non-infected PCCs treated with conditioned medium. Bars show mean ± SEM (*n* = 5) with a one-way ANOVA and a Holm-Sidak’s multiple comparison test (**p < 0.01, *p < 0.05). **h** Conditioned media of HSV-1(17^+^)Lox infected and control PCCs were collected 20 hpi, filtered and supplemented with either FGFR-inhibitor PD173074 (200 nM) or inhibitor vehicle DMSO. Target PCCs were pre-incubated for 2 h with starvation medium containing PD173074 or DMSO before they were incubated with the conditioned media for 30 min. **i** Representative phospho-ERK Western blots of cells treated with conditioned media. **j** Densitometric analysis of ERK phosphorylation in PCCs stimulated with conditioned and supplemented media. Bars show mean ± SEM (*n* = 5) with one-way ANOVA (^+^*p* < 0.05) and Fisher’s LSD post-test with *p < 0.05 and ns indicating non-significance for comparisons between control and HSV-1 CM treated cells and ^#^*p* < 0.05 and ns_#_ for comparisons of PD173074 treated cells with their DMSO control, respectively

Astrocytes are the main source cells for neurotrophic factors in the injured brain [23]. We evaluated the source cell type in the mixed primary cortical cultures by GFAP and FGF-9 immunocytochemistry (Fig. 3a). Indeed, the astrocytes within HSV-1-infected primary cortical cells expressed more FGF-9 compared to control cells which displayed a basic expression only. FGF-9 is secreted via the endoplasmic reticulum and the Golgi apparatus [49]. Accordingly, the HSV-1 induced FGF-9 expression localized to cytoplasmic puncta in a polarized manner (Fig. 3a). However, protein amounts of single FGFs in the medium supernatant were below detection limits for biochemical methods. Indeed, we were not able to detect FGF by mass spectrometry or Western blot with or without purification. However, FGFs effectively display biological activity on living cells and bind to their cognate receptors at only 0.01 ng/ml [50]. We took advantage of this high detection sensitivity of living cells for FGF ligands which commonly activate the mitogen-activated protein kinase (MAPK/ERK) and the serine/threonine-protein kinase Akt. Thus, we collected conditioned media of HSV-1-infected and mock-infected cultures at different time points post infection (30 min, 4 h, and 8 h) (Fig. 3b). Virions were removed by filtration, which was confirmed by plaque assays (Additional file 1: Figure S3). The filtrate was applied to naive cells to measure the cumulative paracrine activity of several FGFs at the same time (Fig. 3b). Both cultures, the source and the target culture, were subsequently lyzed for Western blot analysis (Fig. 3c). ERK and Akt became activated in inoculated cortical cultures with ERK phosphorylation occurring early and Akt phosphorylation with delayed kinetics followed by a downregulation at 8 hpi (Fig. 3d, e). This activity profile corresponds to the transient activation of astrocytes in HSV-1-infected PCCs (Fig. 1) and could be caused by cell intrinsic or extrinsic mechanisms. When transferred to non-infected PCCs, the conditioned media induced ERK-phosphorylation only (Fig. 3f, g). This indicates that HSV-1 infected PCCs secrete a ligand with paracrine activity able to activate the ERK pathway. Notably, ERK phosphorylation in the target cells increased with medium conditioned for longer times from cultures of HSV-1-infected source cells (Fig. 3f). This indicates accumulation of FGFs within the conditioned medium.

To elucidate whether responses of target cells was specific for activation by FGFs, experiments were repeated with minor modifications: Conditioned medium of HSV-1-infected PCCs was collected and filtered and supplemented with nanomolar concentrations of the FGF-receptor inhibitor PD173074 or DMSO only (Fig. 3h). PD173074 is a specific inhibitor of all four FGF-receptors [51] thereby inhibiting all possible FGF ligands irrespective of their receptor preference. Thus, PD173074 inhibits the cumulative effects of several ligands at the same time maximizing the sensitivity of the assay. After 30-min incubation, the target cells were lyzed and the phospho-ERK signal was detected by Western blots which were subsequently quantified (Fig. 3i, j). Again, conditioned medium (CM) from HSV-1-infected PCCs induced a significantly stronger phospho-ERK signal compared to control CM (Fig. 3j). Supplementation of the media with the FGF-receptor inhibitor PD173074 reduced the ERK-activity in both, control and HSV-1 CM treated cells, below that of the DMSO control level (Fig. 3j). This indicates an endogenous FGF ligand production in mock-infected as well as HSV-1-infected cells. These data are supported by our transcript analyses in HSV-1 and mock-infected cells, displaying FGF ligand production in both conditions (Fig. 2 and Additional file 1: Figure S1). Strikingly, there was no difference between HSV-1 and mock-infected conditioned media in their potential to induce phospho-ERK when they were both supplemented with PD173074 (Fig. 3j). Moreover, PD173074 significantly reduced the paracrine activity of HSV-1 CM. In contrast, this effect was not significant in mock-infected control CM (Fig. 3j). Altogether, this demonstrates that FGFs cause the increase of paracrine neurotrophic activity upon HSV-1 infection.

### The FGF response is specific for fully functional HSV-1 in CNS cells

**Fig. 4:**
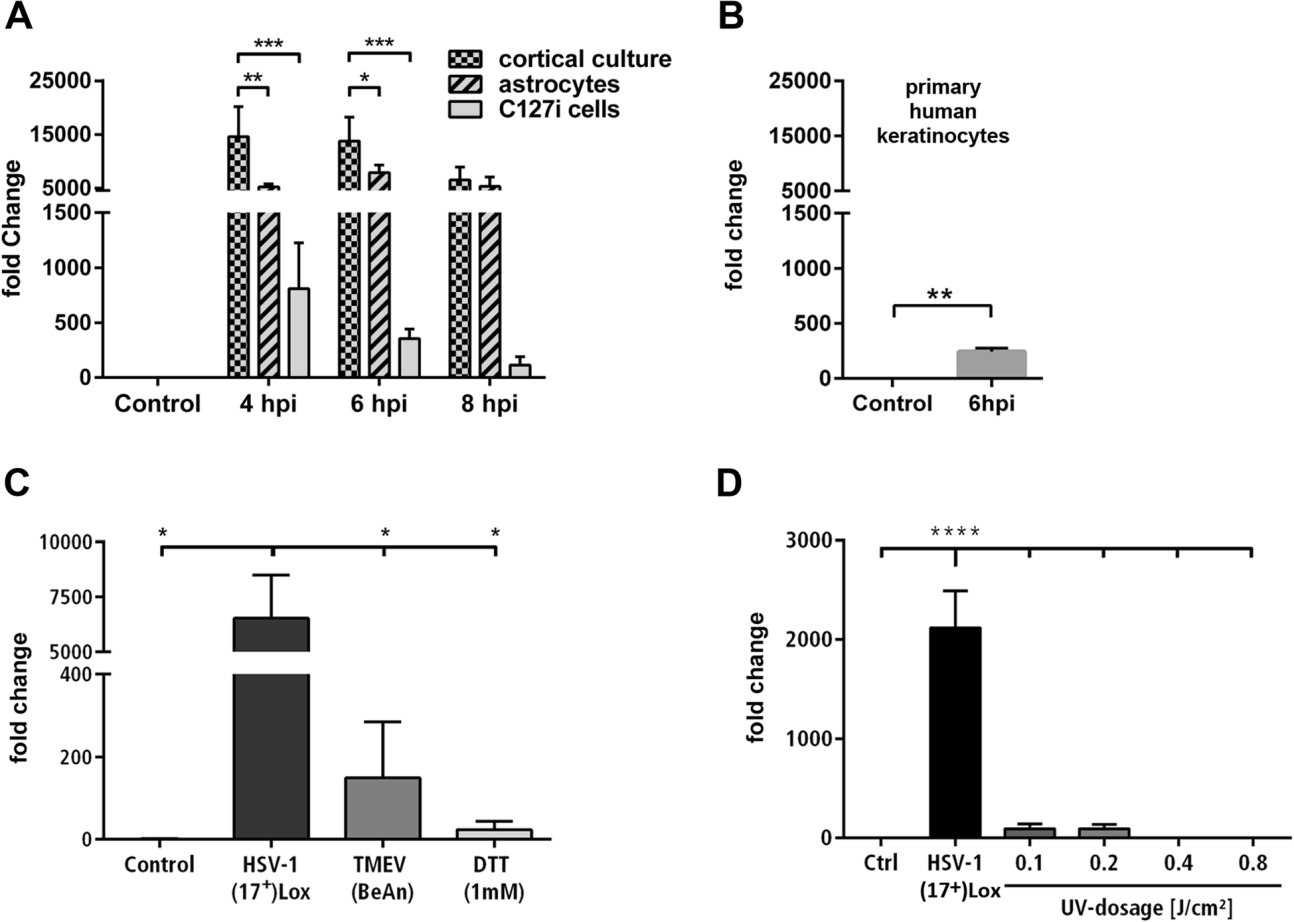
FGF-induction is specific to CNS cells and depends on a functional HSV-1. **a** FGF-4 expression of PCCs, astrocytes, and murine mammary gland epithelial cell line (C127i) infected with HSV-1(17^+^)Lox (MOI 10). **b** FGF-4 expression of mock-infected control primary human keratinocytes compared to HSV-1(17^+^)Lox infected primary human keratinocytes (MOI 10, 6 hpi). Mean ± SEM (*n* = 3); two-way ANOVA with Holm-Sidak’s multiple comparison test (****p < 0.0001, ***p < 0.001, **p < 0.01, *p < 0.05). **c** FGF-4 expression in PCCs infected with HSV-1(17^+^)Lox (MOI 10), Theiler’s murine encephalomyelitis virus (TMEV, BeAn, MOI 10) or treated with dithiothreitol (DTT, 1 mM) 6 h after infection or treatment. **d** Fold change of FGF-4 mRNA in PCCs infected with untreated or UV-inactivated HSV-1(17^+^)Lox. All bars show mean ± SEM (*n* = 3) with Student’s *t* test (**b**), two-way ANOVA (**a**), and one-way ANOVA (**c**–**d**) followed by a Holm-Sidak’s multiple comparison test (*p < 0.05, **p < 0.01, ***p < 0.001, ****p < 0.0001)

Next, we tested the cell type specificity of the FGF response using FGF-4 mRNA levels as readout since it was the most abundant FGF which was regulated in a switch-on like manner (Fig. 2). In addition to neurons and astrocytes, HSV-1 infects epithelial cells and fibroblasts. Next to a primary murine astrocyte culture, we included the murine mammary gland epithelial cell line (C127i) in our analysis. HSV-1-infected PCCs expressed higher levels of FGF-4 mRNA compared to astrocytes, however, both responded with a strong FGF-4 induction (Fig. 4a). In comparison, a marginal amount of FGF-4 mRNA was detected in infected epithelial cells, thus indicating a tissue and cell-specific response. Moreover, we tested HSV-1-infected human primary keratinocytes for FGF-4 expression. Similar to the murine C127i cell-line, an HSV-1 infection induced a low but significant FGF-4 expression (Fig. 4b). We further evaluated whether FGF-4 expression is a general response to cell stress, viral infections, or more specific to HSV-1. We infected PCCs with HSV-1, with the RNA-virus Theiler’s murine encephalomyelitis virus (TMEV) or treated the cells with the global stress inducer dithiothreitol (DTT) (Fig. 4c). HSV-1 infection significantly induced the production of FGF-4 mRNA, whereas TMEV infection and DTT treatment displayed a basal FGF-4 expression only (Fig. 4c). We characterized the HSV-1 contribution to the FGF-4 response inoculating PCCs with UV-inactivated HSV-1 (Fig. 4d). We used different doses of UV-light for the inactivation of HSV-1(17^+^)Lox and incubated those virions together with PCCs. The degree of inactivation was monitored by qRT-PCR of immediate-early, early, and late HSV-1 transcripts. HSV-1 treated at low dose of UV-light was still able to infect the cells and induce late viral transcript production (Additional file 1: Figure S4). Interestingly, this treatment reduced the HSV-1-mediated FGF-4 expression to a marginal level—a slightly damaged HSV-1 was not capable to efficiently induce the FGF-4 mRNA. This indicates that the FGF-response is induced by the virus rather than being an antiviral host-response. Indeed, we could not detect FGF-4 induction in PCCs treated with diverse agonists of innate immunity (Additional file 1: Figure S5). Moreover, we included PCCs from STING or MyD88 knock-out mice which are elements of innate immune DNA sensing and downstream, respectively, pathways. However, the FGF-4 on-switch was not impaired in those cells (Additional file 1: Figure S5).

### The viral ICP0 protein mediates the FGF-response in CNS cells

**Fig. 5:**
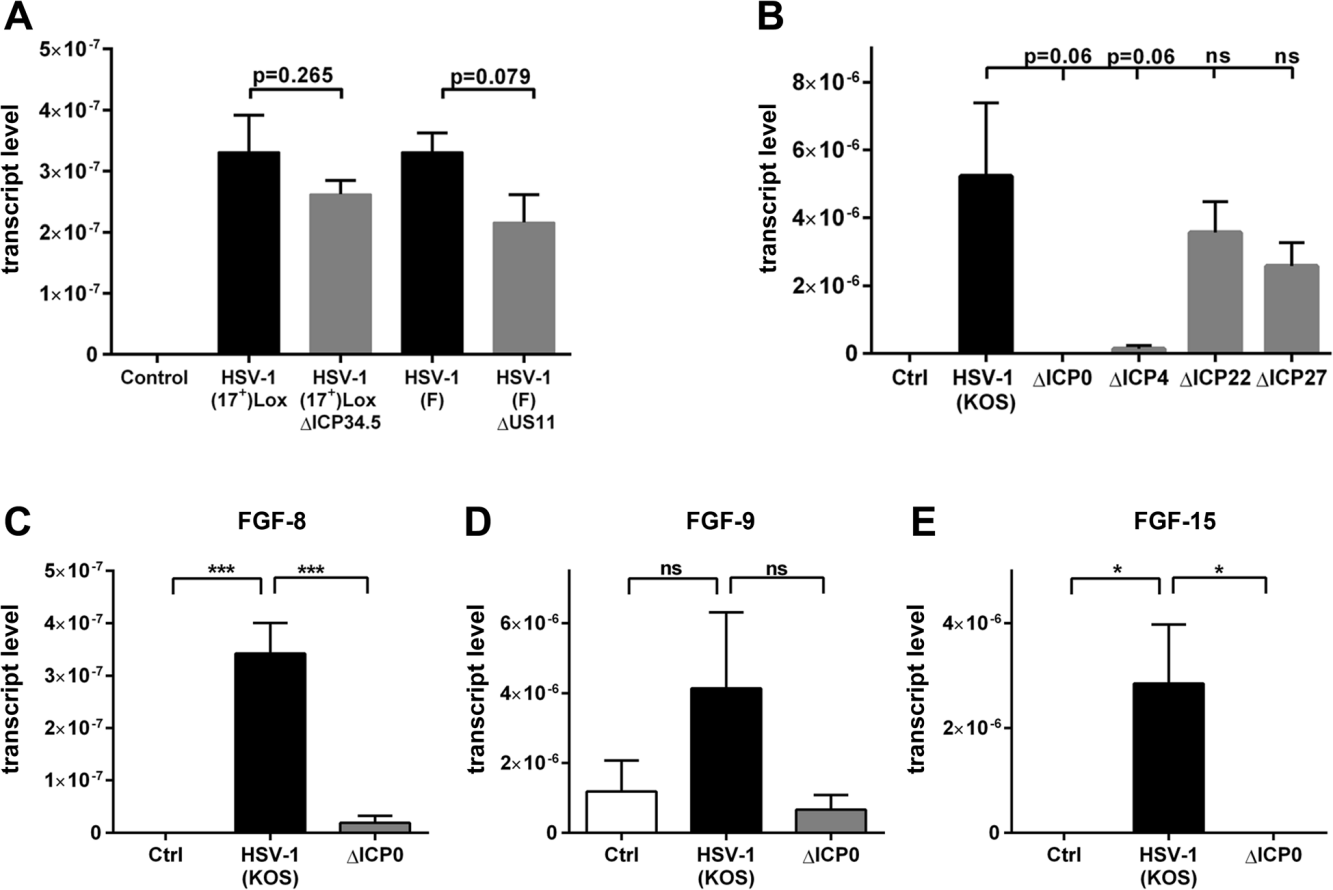
ICP0 deficient HSV-1 is not able to induce an FGF-response. **a** FGF-4 mRNA expression of PCCs which were infected with deletion mutants for neurovirulence factor ICP34.5 or RNA binding protein US11 (MOI 10, 6 hpi). **b** PCCs were infected with HSV-1 (KOS) as well as HSV-1 KOS knockout strains lacking ICP0, 4, 22, and 27 (MOI = 10, 6 hpi). **c**–**e** FGF transcripts of HSV-1 (KOS) infected PCCs compared to the knockout KOS strain without ICP0 (MOI = 10, 6 hpi). All bars show mean ± SEM (*n* = 3) with a one-way ANOVA followed by a Holm-Sidak’s multiple comparison test (*p < 0.05, ***p < 0.001)

We therefore hypothesized that the virus actively induces FGF production and characterized the molecular mechanism using HSV-1 knockout strains. Considering the neurotropism of HSV-1 and the role of FGFs in the neuronal system, we tested a mutant which was deficient for the non-essential neurovirulence factor ICP34.5 [32]. ICP34.5-deficient HSV-1 mutants can replicate in epithelial cells but lack the ability to spread in the nervous system [52]. The other mutated HSV-1 strain lacked the non-essential RNA-binding protein US11, which associates with 60S ribosomal subunits and regulates initiation of viral transcription [33]. These deletion mutants resulted in a similar induction of FGF-4 expression compared to the corresponding parental strains (Fig. 5a). Thus, we included more mutants based on the parental KOS strain. FGF-4 mRNA levels were the same in PCCs infected with parental wild-type HSV-1 strain and viruses lacking ICP22 and ICP27. Interestingly, ICP4-deficient viruses displayed a strong reduction in FGF-4 expression while the switch-on mechanism was completely abolished in PCCs infected with ICP0-deficient viruses (Fig. 5b). Next to FGF-4, we found an upregulation of FGF-8 and FGF-15 in a switch-on like mechanism and a tendency for FGF-9 induction (Fig. 2). We tested the HSV-1 induction of those FGFs for their dependence on ICP0. Similar to FGF-4, FGF-8 and FGF-15 expression was induced by HSV-1 KOS strain only when comprising ICP0 (Fig. 5c–e). This indicates that HSV-1 uses the same ICP0 dependent switch-on mechanism for the induction of FGF-4, FGF-8, and FGF-15.

## Discussion

Here, we showed that cortical brain cells react to HSV-1 infections with an altered pattern of FGF expression. The expression of several FGFs was dramatically upregulated in HSV-1-infected cortical cells consistent with an FGFR-dependent ERK activation in neighboring cells. Notably, human primary keratinocytes displayed a low but significant FGF-ligand expression. However, the biological relevance of this finding is unclear so far. HSV-1-infected astrocytes transiently changed their morphology becoming less branched but larger. This is a clear indication of astrocyte activation, since reactive astrocytes become hypertrophic with an increased expression of intermediate filament proteins [53, 54]. Recently, activated astrocytes were classified into A1 and A2 phenotypes [20]. A1 reactive astrocytes produce cytokines and often form a compact glial scar to limit tissue damage, but inhibit axon regeneration and cell survival [55]. On the other hand, A2 astrocytes act in a repair environment and promote regeneration by secretion of growth factors [55, 56]. HSV-1-infected cortical cultures displayed an induction of TNF-α production concomitant with a morphologically detectable astrocyte-activation. Activated microglia secrete TNF-α to promote the A1 profile of astrocytes [20]. Consistently, HSV-1-inoculated cortical cultures expressed TNF-α as well as the A1 marker CXCL10. However, at the same time, we could detect the A2-marker Cox2. A possible explanation is an unconventional astrocyte-activation, a mixed cell population of A1 and A2 cells or astrocytes displaying a continuum between A1 and A2 subtype.

Using conditioned media of HSV-1-infected cells, we could show enhanced paracrine activity of the neurotrophic FGF-system. Neurotrophic factors support the recovery from tissue damage and suppress further neurotoxicity caused by ongoing release of pro-inflammatory mediators and glial scar formation [16, 57]. Various studies demonstrated the presence of neurotrophic factors in CNS disorders induced by infections or other inflammatory diseases [16, 58]. However, the high expression level of FGF-4 in response to HSV-1 infection compared to another virus or cellular stress indicates that this effect may be specific for HSV-1. TMEV or DTT yet induced a low FGF-4 signal indicating a basal reaction strongly amplified by HSV-1 infections. While we could not find an influence of innate sensing pathways on FGF-expression, only a fully functional virus comprising ICP0 could efficiently induce FGF expression. Together, this indicates that HSV-1 actively induces the FGF-response in an ICP0-dependent manner.

ICP0 is essential for the FGF switch providing a potential link for mechanistic insights. However, a detailed evaluation of the mechanisms will be part of future studies. ICP0 is an immediate early protein which is able to transactivate immediate early, early, and late HSV-1 genes enhancing viral replication [36]. Thereby, it inactivates defense mechanisms of the host at several regulatory levels [59]. ICP0 is an E3 ubiquitin ligase with a nuclear localization sequence facilitating the proteasomal degradation of its target proteins [60]. It may directly induce the degradation of the IFN-γ-inducible protein Ifi16, a DNA-sensor for innate immunity [61, 62], and MyD88 as part of the Toll-like receptor signaling [63]. Another function of ICP0 is its indirect interference with gene-transcription. ICP0 induces the degradation of the ubiquitin-specific protease 7 (USP7) [64] thereby destabilizing transcriptional repressors such as the polycomb repressive complex [65]. Interestingly, the polycomb repressive complex inhibits FGF-8 expression [66], one of the FGFs which was upregulated upon HSV-1 infection. Alternatively, FGF expression may be induced via ICP0-mediated degradation of promyelocytic leukemia protein (PML) [67] which also suppresses FGF-8 expression [68].

There are 22 FGF-family members with four canonical FGF-receptors in humans and mice [25, 69]. FGF ligands can be divided into subfamilies by their mode of action as well as their phylogeny. FGFs-11/12/13/14 are not secreted and act intracellularly while endocrine FGFs-15/21/23 signal via long-distances. All other FGFs signal in a para- or autocrine manner [25]. Here, we report an enhanced expression of 8 FGF ligands upon HSV-1 infection among which there was no FGF belonging to the intracellular subfamily. The only endocrine FGF was FGF-15 which promotes neuronal differentiation in cortical development [70]. The other upregulated paracrine FGFs were FGF-3 as the only member of the FGF-7 subfamily, FGF-8 as the only member of the FGF-8 subfamily, FGF-9 and 20 which belong to the FGF-9 subfamily, and the complete FGF-4 subfamily (FGF-4, 5, and 6). Neurotrophic activity has been reported for FGF-8, 9, and 20 [71–73] and their upregulation in HSV-1-infected PCCs may protect neurons from apoptosis. FGF-6 is expressed in the brain with a yet undefined role [74]. FGF-5 may have some neurotrophic activity in vitro [75], although this is less clear in vivo [76]. FGF-4 mRNA is expressed at the rostral migratory region and the subventricular zone. The protein induces neuronal progenitor proliferation and neuronal differentiation indicating a role in neurogenesis [77].

Besides neurons, glial cells such as astrocytes are important targets for FGF-signaling in the CNS. An astrocyte-specific depletion of the FGF-receptors induced a de novo astrogliosis in healthy brains and enhanced the astrogliosis after brain damage leading to an enlarged glial scar. This indicates an inhibitory effect of FGF signaling on proliferation during inflammatory astrocyte-activation [78]. Interestingly, astrocytes which were incubated with FGF-8 became migratory and hypertrophic [27]—reminiscent of HSV-1-infected PCCs which produce more FGF-8 and comprise enlarged astrocytes. On the one hand, FGF-signaling suppresses an inflammatory-astrogliosis, but on the other hand it induces a migratory, hypertrophic phenotype which may relate to A2 astrocytes. Thus FGFs may mediate the important A1 to A2 shift thereby promoting repair over removal of damaged cells. Considering this, FGFs secreted from HSV-1-infected CNS cells may be anti-inflammatory via astrocytic FGFR activation and promote regeneration and repair via neuronal FGFR activation. Whether this is beneficial in the context of HSV-1 CNS infections or promotes detrimental viral spread needs to be resolved in future studies.

## Conclusions

HSV-1 induces FGF production in primary cortical brain cultures which depends on the viral protein ICP0 and induces neurotrophic signaling in neighboring cells.

## Supplementary information

**Additional file 1: Figure S1.** mRNAs of housekeeping genes and some FGFs become down-regulated in HSV-1 infected PCCs. A, Negatively regulated FGF mRNAs in HSV-1(17^+^)Lox (MOI 10) infected PCCs, mean ± SEM of technical duplicates. B, mRNAs of housekeeping genes GAPDH and PPIA in PCCs infected with HSV-1(17^+^)LOX. C, mRNA levels of GDNF and NGF in infected. Mean ± SEM, n=3, two-way ANOVA, Holm-Sidak‘s multiple comparison test (* *p*<0.05, ** *p*<0.01, *** *p*<0.001). **Figure S2.** FGF-4 is correctly spliced and expressed in HSV-1 infected PCCs. HSV-1 randomly provokes a dysregulation of transcription termination producing non-translated transcripts including introns [79]. A, Two primer sets were used for amplification of spliced and non-spliced FGF-4 transcripts in qRT-PCR (black arrows) and conventional RT-PCR (red arrows). B, cDNA and RNA from control and HSV-1(17^+^)Lox infected PCCs (MOI 10, 6 hpi) was used with primers able to amplify intron sequences (red arrows in A). NTC = non template control. C, Fold change of FGF-4 mRNA in HSV-1(17^+^)Lox infected PCCs (MOI10). Mean ± SEM, one-way ANOVA, Holm-Sidak‘s multiple comparison test (****p>0.0001). **Figure S3.** Filtration of HSV-1 conditioned medium removes viral particles. The media of HSV-1 infected cells were filtered followed by plaque assays. We could observe plaques in non-filtered media (detection limit 40 PFU/ml), but no plaques in cells incubated with filtered, HSV-1 conditioned medium. Mean ± SEM, n=5. **Figure S4.** Increasing doses of UV-radiation de-activate HSV-1. Transcript level of different viral genes in PCCs infected with untreated or UV-inactivated HSV-1(17^+^)Lox (MOI 10, 4 hpi). Mean from three pooled replicates. **Figure S5.** FGF-4 responses are not triggered by MyD88 dependent TLRs or cGAS/STING innate sensing mechanisms. A, PCCs were incubated with TLR agonists for 6 hours and FGF-4 mRNA levels were quantified. Mean, n=1. B, PCCs derived from STING or MyD88 knock-out mice infected with HSV-1 (MOI 10). Mean ± SEM, n=5, one-way ANOVA, Holm-Sidak‘s multiple comparison test (*** *p* < 0.001, ** *p* < 0.01, **** *p* < 0.0001). **Table S1.** PCR-primer sequences for the detection of murine transcripts or HSV-1 transcripts.

## Data Availability

All data generated or analyzed during this study are included in this published article and its supplementary information files.

## Abbreviations

BNDF: Brain-derived neurotrophic factor
CNS: Central nervous system
CNTF: Ciliary neurotrophic factor
DIV: Day in vitro
DTT: Dithiothreitol
FGF: Fibroblast growth factor
FGFR: Fibroblast growth factor receptor
GAPDH: Glyceraldehyde 3-phosphate dehydrogenase
GDNF: Glial derived neurotrophic factor
GFP: Green fluorescent protein
HIV: Human immunodeficiency virus
HSE: HSV-1 encephalitis
HSV-1: Herpes simplex virus-1
ICP: HSV-infected cell protein
MAPK: Mitogen-activated protein kinase
MOI: Multiplicity of infection
NGF: Nerve growth factor
NT: Eurotrophic
PCC: Primary murine cortical cell culture
PLL: Poly-l-lysine
PML: Promyelocytic leukemia protein
PPIA: Peptidylprolyl isomerase A
RIN: RNA integrity number
TLR: Toll-like receptor
TMEV: Theiler’s murine encephalomyelitis virus
USP7: Ubiquitin specific protease 7

## References

[1] Smith MG, Lennette EH, Reames HR (1941). Isolation of the virus of herpes simplex and the demonstration of intranuclear inclusions in a case of acute encephalitis. Am J Pathol.

[2] Boucher A, Herrmann JL, Morand P, Buzele R, Crabol Y, Stahl JP, Mailles A (2017). Epidemiology of infectious encephalitis causes in 2016. Med Mal Infect.

[3] McGrath N, Anderson NE, Croxson MC, Powell KF (1997). Herpes simplex encephalitis treated with acyclovir: diagnosis and long term outcome. J Neurol Neurosurg Psychiatry.

[4] Moon SM, Kim T, Lee EM, Kang JK, Lee SA, Choi SH (2014). Comparison of clinical manifestations, outcomes and cerebrospinal fluid findings between herpes simplex type 1 and type 2 central nervous system infections in adults. J Med Virol.

[5] O'Sullivan CE, Aksamit AJ, Harrington JR, Harmsen WS, Mitchell PS, Patel R (2003). Clinical spectrum and laboratory characteristics associated with detection of herpes simplex virus DNA in cerebrospinal fluid. Mayo Clin Proc.

[6] Kapur N, Barker S, Burrows EH, Ellison D, Brice J, Illis LS, Scholey K, Colbourn C, Wilson B, Loates M (1994). Herpes simplex encephalitis: long term magnetic resonance imaging and neuropsychological profile. J Neurol Neurosurg Psychiatry.

[7] Smith JS, Robinson NJ (2002). Age-specific prevalence of infection with herpes simplex virus types 2 and 1: a global review. J Infect Dis.

[8] Whitley RJ (2006). Herpes simplex encephalitis: adolescents and adults. Antiviral Res.

[9] Smith G (2012). Herpesvirus transport to the nervous system and back again. Annu Rev Microbiol.

[10] Knipe DM, Cliffe A (2008). Chromatin control of herpes simplex virus lytic and latent infection. Nat Rev Microbiol.

[11] Russell TA, Tscharke DC (2016). Lytic promoters express protein during herpes simplex virus latency. PLoS Pathog.

[12] Menendez CM, Carr DJJ (2017). Defining nervous system susceptibility during acute and latent herpes simplex virus-1 infection. J Neuroimmunol.

[13] Roizman B, Whitley RJ (2013). An inquiry into the molecular basis of HSV latency and reactivation. Annu Rev Microbiol.

[14] Tan IL, McArthur JC, Venkatesan A, Nath A (2012). Atypical manifestations and poor outcome of herpes simplex encephalitis in the immunocompromised. Neurology.

[15] Hjalmarsson A, Blomqvist P, Skoldenberg B (2007). Herpes simplex encephalitis in Sweden, 1990-2001: incidence, morbidity, and mortality. Clin Infect Dis.

[16] Fields J, Dumaop W, Langford TD, Rockenstein E, Masliah E (2014). Role of neurotrophic factor alterations in the neurodegenerative process in HIV associated neurocognitive disorders. J Neuroimmune Pharmacol.

[17] Sakai S, Shichita T. Inflammation and neural repair after ischemic brain injury. Neurochem Int. 2018. 10.1016/j.neuint.2018.10.013 30342960

[18] Finnie JW (2013). Neuroinflammation: beneficial and detrimental effects after traumatic brain injury. Inflammopharmacology.

[19] da Silva ML, Simon D, Regner A. Neurotrauma: the crosstalk between neurotrophins and inflammation in the acutely injured brain. Int J Mol Sci. 2017;18. 10.3390/ijms18051082 PMC5454991 28524074

[20] Liddelow SA, Guttenplan KA, Clarke LE, Bennett FC, Bohlen CJ, Schirmer L, Bennett ML, Munch AE, Chung WS, Peterson TC (2017). Neurotoxic reactive astrocytes are induced by activated microglia. Nature.

[21] Zamanian JL, Xu L, Foo LC, Nouri N, Zhou L, Giffard RG, Barres BA (2012). Genomic analysis of reactive astrogliosis. J Neurosci.

[22] Burda JE, Bernstein AM, Sofroniew MV (2016). Astrocyte roles in traumatic brain injury. Exp Neurol.

[23] Goss JR, O'Malley ME, Zou L, Styren SD, Kochanek PM, DeKosky ST (1998). Astrocytes are the major source of nerve growth factor upregulation following traumatic brain injury in the rat. Exp Neurol.

[24] Mufson EJ, Kroin JS, Sendera TJ, Sobreviela T (1999). Distribution and retrograde transport of trophic factors in the central nervous system: functional implications for the treatment of neurodegenerative diseases. Prog Neurobiol.

[25] Itoh N, Ornitz DM (2008). Functional evolutionary history of the mouse Fgf gene family. Dev Dyn.

[26] Gehrmann J, Lannes-Vieira J, Wekerle H (1996). Differential expression of fibroblast growth factor-2 and receptor by glial cells in experimental autoimmune encephalomyelitis (EAE). Glia.

[27] Kang K, Lee SW, Han JE, Choi JW, Song MR (2014). The complex morphology of reactive astrocytes controlled by fibroblast growth factor signaling. Glia.

[28] Tang MM, Lin WJ, Pan YQ, Li YC (2018). Fibroblast growth factor 2 modulates hippocampal microglia activation in a neuroinflammation induced model of depression. Front Cell Neurosci.

[29] Hausott B, Klimaschewski L (2019). Promotion of peripheral nerve regeneration by stimulation of the extracellular signal-regulated kinase (ERK) pathway. Anat Rec (Hoboken).

[30] Zhang Y, Yeruva L, Marinov A, Prantner D, Wyrick PB, Lupashin V, Nagarajan UM (2014). The DNA sensor, cyclic GMP-AMP synthase, is essential for induction of IFN-beta during Chlamydia trachomatis infection. J Immunol.

[31] Adachi O, Kawai T, Takeda K, Matsumoto M, Tsutsui H, Sakagami M, Nakanishi K, Akira S (1998). Targeted disruption of the MyD88 gene results in loss of IL-1- and IL-18-mediated function. Immunity.

[32] Nygardas M, Paavilainen H, Muther N, Nagel CH, Roytta M, Sodeik B, Hukkanen V (2013). A herpes simplex virus-derived replicative vector expressing LIF limits experimental demyelinating disease and modulates autoimmunity. PLoS One.

[33] Roller RJ, Monk LL, Stuart D, Roizman B (1996). Structure and function in the herpes simplex virus 1 RNA-binding protein U(s)11: mapping of the domain required for ribosomal and nucleolar association and RNA binding in vitro. J Virol.

[34] Snijder B, Sacher R, Ramo P, Liberali P, Mench K, Wolfrum N, Burleigh L, Scott CC, Verheije MH, Mercer J (2012). Single-cell analysis of population context advances RNAi screening at multiple levels. Mol Syst Biol.

[35] Devadas D, Koithan T, Diestel R, Prank U, Sodeik B, Dohner K (2014). Herpes simplex virus internalization into epithelial cells requires Na+/H+ exchangers and p21-activated kinases but neither clathrin- nor caveolin-mediated endocytosis. J Virol.

[36] Cai WZ, Schaffer PA (1989). Herpes simplex virus type 1 ICP0 plays a critical role in the de novo synthesis of infectious virus following transfection of viral DNA. J Virol.

[37] Long MC, Leong V, Schaffer PA, Spencer CA, Rice SA (1999). ICP22 and the UL13 protein kinase are both required for herpes simplex virus-induced modification of the large subunit of RNA polymerase II. J Virol.

[38] DeLuca NA, Schaffer PA (1987). Activities of herpes simplex virus type 1 (HSV-1) ICP4 genes specifying nonsense peptides. Nucleic Acids Res.

[39] Rice SA, Knipe DM (1990). Genetic evidence for two distinct transactivation functions of the herpes simplex virus alpha protein ICP27. J Virol.

[40] Kummerfeld M, Meens J, Haas L, Baumgartner W, Beineke A (2009). Generation and characterization of a polyclonal antibody for the detection of Theiler’s murine encephalomyelitis virus by light and electron microscopy. J Virol Methods.

[41] Janssen S, Schlegel C, Gudi V, Prajeeth CK, Skripuletz T, Trebst C, Stangel M (2015). Effect of FTY720-phosphate on the expression of inflammation-associated molecules in astrocytes in vitro. Mol Med Rep.

[42] Seele Jana, Nau Roland, Prajeeth Chittappen, Stangel Martin, Valentin-Weigand Peter, Seitz Maren (2016). Astrocytes Enhance Streptococcus suis-Glial Cell Interaction in Primary Astrocyte-Microglial Cell Co-Cultures. Pathogens.

[43] Dohner K, Wolfstein A, Prank U, Echeverri C, Dujardin D, Vallee R, Sodeik B (2002). Function of dynein and dynactin in herpes simplex virus capsid transport. Mol Biol Cell.

[44] Ratzka A, Baron O, Grothe C (2011). FGF-2 deficiency does not influence FGF ligand and receptor expression during development of the nigrostriatal system. PLoS One.

[45] Ojala PM, Sodeik B, Ebersold MW, Kutay U, Helenius A (2000). Herpes simplex virus type 1 entry into host cells: reconstitution of capsid binding and uncoating at the nuclear pore complex in vitro. Mol Cell Biol.

[46] Carpenter AE, Jones TR, Lamprecht MR, Clarke C, Kang IH, Friman O, Guertin DA, Chang JH, Lindquist RA, Moffat J (2006). CellProfiler: image analysis software for identifying and quantifying cell phenotypes. Genome Biol.

[47] Beaudoin GM, Lee SH, Singh D, Yuan Y, Ng YG, Reichardt LF, Arikkath J (2012). Culturing pyramidal neurons from the early postnatal mouse hippocampus and cortex. Nat Protoc.

[48] Spencer CA, Dahmus ME, Rice SA (1997). Repression of host RNA polymerase II transcription by herpes simplex virus type 1. J Virol.

[49] Revest JM, DeMoerlooze L, Dickson C (2000). Fibroblast growth factor 9 secretion is mediated by a non-cleaved amino-terminal signal sequence. J Biol Chem.

[50] Zhu H, Duchesne L, Rudland PS, Fernig DG (2010). The heparan sulfate co-receptor and the concentration of fibroblast growth factor-2 independently elicit different signalling patterns from the fibroblast growth factor receptor. Cell Commun Signal.

[51] Mohammadi M, Froum S, Hamby JM, Schroeder MC, Panek RL, Lu GH, Eliseenkova AV, Green D, Schlessinger J, Hubbard SR (1998). Crystal structure of an angiogenesis inhibitor bound to the FGF receptor tyrosine kinase domain. EMBO J.

[52] Chou J, Kern ER, Whitley RJ, Roizman B (1990). Mapping of herpes simplex virus-1 neurovirulence to gamma 134.5, a gene nonessential for growth in culture. Science.

[53] Koyama Y (2014). Signaling molecules regulating phenotypic conversions of astrocytes and glial scar formation in damaged nerve tissues. Neurochem Int.

[54] Sofroniew MV, Vinters HV (2010). Astrocytes: biology and pathology. Acta Neuropathol.

[55] Liberto CM, Albrecht PJ, Herx LM, Yong VW, Levison SW (2004). Pro-regenerative properties of cytokine-activated astrocytes. J Neurochem.

[56] Ridet JL, Malhotra SK, Privat A, Gage FH (1997). Reactive astrocytes: cellular and molecular cues to biological function. Trends Neurosci.

[57] Ye L, Yang Y, Zhang X, Cai P, Li R, Chen D, Wei X, Zhang X, Xu H, Xiao J, et al. The role of bFGF in the excessive activation of astrocytes is related to the inhibition of TLR4/NFkappaB signals. Int J Mol Sci. 2015;17. 10.3390/ijms17010037 PMC4730282 26729092

[58] Kizawa-Ueda M, Ueda A, Kawamura N, Ishikawa T, Mutoh E, Fukuda Y, Shiroki R, Hoshinaga K, Ito S, Asakura K, Mutoh T (2011). Neurotrophin levels in cerebrospinal fluid of adult patients with meningitis and encephalitis. Eur Neurol.

[59] Lanfranca MP, Mostafa HH, Davido DJ (2014). HSV-1 ICP0: An E3 ubiquitin ligase that counteracts host intrinsic and innate immunity. Cells.

[60] Everett RD (2000). ICP0 induces the accumulation of colocalizing conjugated ubiquitin. J Virol.

[61] Orzalli MH, DeLuca NA, Knipe DM (2012). Nuclear IFI16 induction of IRF-3 signaling during herpesviral infection and degradation of IFI16 by the viral ICP0 protein. Proc Natl Acad Sci U S A.

[62] Cuchet-Lourenco D, Anderson G, Sloan E, Orr A, Everett RD (2013). The viral ubiquitin ligase ICP0 is neither sufficient nor necessary for degradation of the cellular DNA sensor IFI16 during herpes simplex virus 1 infection. J Virol.

[63] van Lint AL, Murawski MR, Goodbody RE, Severa M, Fitzgerald KA, Finberg RW, Knipe DM, Kurt-Jones EA (2010). Herpes simplex virus immediate-early ICP0 protein inhibits Toll-like receptor 2-dependent inflammatory responses and NF-kappaB signaling. J Virol.

[64] Boutell C, Canning M, Orr A, Everett RD (2005). Reciprocal activities between herpes simplex virus type 1 regulatory protein ICP0, a ubiquitin E3 ligase, and ubiquitin-specific protease USP7. J Virol.

[65] de Bie P, Zaaroor-Regev D, Ciechanover A (2010). Regulation of the polycomb protein RING1B ubiquitination by USP7. Biochem Biophys Res Commun.

[66] Kumar S, Duester G (2014). Retinoic acid controls body axis extension by directly repressing Fgf8 transcription. Development.

[67] Perusina Lanfranca M, Mostafa HH, Davido DJ (2013). Two overlapping regions within the N-terminal half of the herpes simplex virus 1 E3 ubiquitin ligase ICP0 facilitate the degradation and dissociation of PML and dissociation of Sp100 from ND10. J Virol.

[68] Kwong WH, Tang MK, Yew DT, Chan JY, Cai DQ, Tong WM, Lee KK (2001). Fibroblast growth factor-8b-stimulated myogenic cell proliferation is suppressed by the promyelocytic leukemia gene. Biol Signals Recept.

[69] Itoh N, Ornitz DM (2004). Evolution of the Fgf and Fgfr gene families. Trends Genet.

[70] Borello U, Cobos I, Long JE, McWhirter JR, Murre C, Rubenstein JL (2008). FGF15 promotes neurogenesis and opposes FGF8 function during neocortical development. Neural Dev.

[71] Tanaka A, Kamiakito T, Hakamata Y, Fujii A, Kuriki K, Fukayama M (2001). Extensive neuronal localization and neurotrophic function of fibroblast growth factor 8 in the nervous system. Brain Res.

[72] Kanda T, Iwasaki T, Nakamura S, Ueki A, Kurokawa T, Ikeda K, Mizusawa H (1999). FGF-9 is an autocrine/paracrine neurotrophic substance for spinal motoneurons. Int J Dev Neurosci.

[73] Ohmachi S, Watanabe Y, Mikami T, Kusu N, Ibi T, Akaike A, Itoh N (2000). FGF-20, a novel neurotrophic factor, preferentially expressed in the substantia nigra pars compacta of rat brain. Biochem Biophys Res Commun.

[74] Ozawa K, Uruno T, Miyakawa K, Seo M, Imamura T (1996). Expression of the fibroblast growth factor family and their receptor family genes during mouse brain development. Brain Res Mol Brain Res.

[75] Hughes RA, Sendtner M, Goldfarb M, Lindholm D, Thoenen H (1993). Evidence that fibroblast growth factor 5 is a major muscle-derived survival factor for cultured spinal motoneurons. Neuron.

[76] McGeachie AB, Koishi K, Imamura T, McLennan IS (2001). Department of A, Structural Biology UoODNZ: Fibroblast growth factor-5 is expressed in Schwann cells and is not essential for motoneurone survival. Neuroscience.

[77] Kosaka N, Kodama M, Sasaki H, Yamamoto Y, Takeshita F, Takahama Y, Sakamoto H, Kato T, Terada M, Ochiya T (2006). FGF-4 regulates neural progenitor cell proliferation and neuronal differentiation. FASEB J.

[78] Kang W, Balordi F, Su N, Chen L, Fishell G, Hebert JM (2014). Astrocyte activation is suppressed in both normal and injured brain by FGF signaling. Proc Natl Acad Sci U S A.

[79] Rutkowski AJ, Erhard F, L'Hernault A, Bonfert T, Schilhabel M, Crump C, Rosenstiel P, Efstathiou S, Zimmer R, Friedel CC, Dolken L (2015). Widespread disruption of host transcription termination in HSV-1 infection. Nat Commun.

