# Supplementary material for: HSV-1 triggers paracrine fibroblast growth factor response from cortical brain cells via immediate-early protein ICP0
Source: J Neuroinflammation. 2019 Dec 2;16:248. doi: 10.1186/s12974-019-1647-5 (PMC6889453; doi:10.1186/s12974-019-1647-5)
Supplement: Supplementary file 1 — Additional file 1: Figure S1. mRNAs of housekeeping genes and some FGFs become down-regulated in HSV-1 infected PCCs. A, Negatively regulated FGF mRNAs in HSV-1(17+)Lox (MOI 10) infected PCCs, mean ± SEM of technical duplicates. B, mRNAs of housekeeping genes GAPDH and PPIA in PCCs infected with HSV-1(17+)LOX. C, mRNA levels of GDNF and NGF in infected. Mean ± SEM, n=3, two-way ANOVA, Holm-Sidak‘s multiple comparison test (* p<0.05, ** p<0.01, *** p<0.001). Figure S2. FGF-4 is correctly spliced and expressed in HSV-1 infected PCCs. HSV-1 randomly provokes a dysregulation of transcription termination producing non-translated transcripts including introns [79]. A, Two primer sets were used for amplification of spliced and non-spliced FGF-4 transcripts in qRT-PCR (black arrows) and conventional RT-PCR (red arrows). B, cDNA and RNA from control and HSV-1(17+)Lox infected PCCs (MOI 10, 6 hpi) was used with primers able to amplify intron sequences (red arrows in A). NTC = non template control. C, Fold change of FGF-4 mRNA in HSV-1(17+)Lox infected PCCs (MOI10). Mean ± SEM, one-way ANOVA, Holm-Sidak‘s multiple comparison test (****p>0.0001). Figure S3. Filtration of HSV-1 conditioned medium removes viral particles. The media of HSV-1 infected cells were filtered followed by plaque assays. We could observe plaques in non-filtered media (detection limit 40 PFU/ml), but no plaques in cells incubated with filtered, HSV-1 conditioned medium. Mean ± SEM, n=5. Figure S4. Increasing doses of UV-radiation de-activate HSV-1. Transcript level of different viral genes in PCCs infected with untreated or UV-inactivated HSV-1(17+)Lox (MOI 10, 4 hpi). Mean from three pooled replicates. Figure S5. FGF-4 responses are not triggered by MyD88 dependent TLRs or cGAS/STING innate sensing mechanisms. A, PCCs were incubated with TLR agonists for 6 hours and FGF-4 mRNA levels were quantified. Mean, n=1. B, PCCs derived from STING or MyD88 knock-out mice infected with HSV-1 (MOI 10). Mean ± SEM, n= [file 12974_2019_1647_MOESM1_ESM.docx]

**SUPPLEMENT**

**Fig. S1:** mRNAs of housekeeping genes and some FGFs become down-regulated in HSV-1 infected PCCs.

**
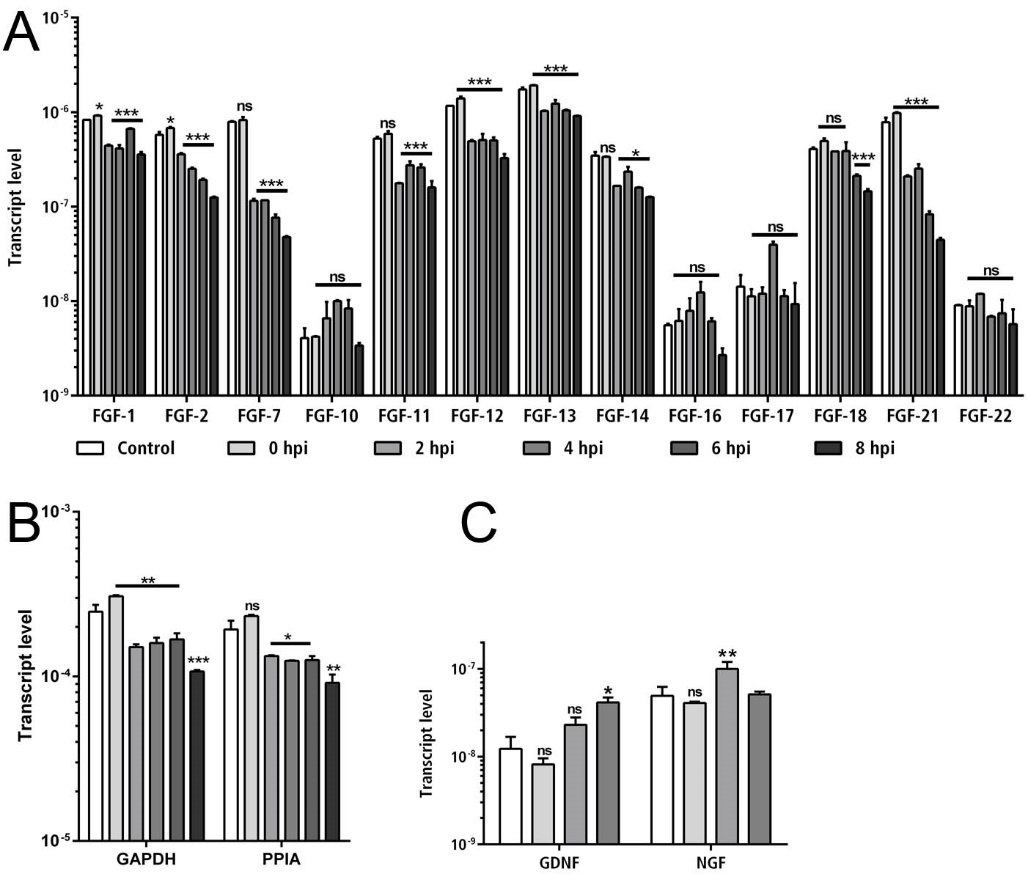
**

**A,** Negatively regulated FGF mRNAs in HSV-1(17^+^)Lox (MOI 10) infected PCCs, mean ± SEM of technical duplicates. **B,** mRNAs of housekeeping genes GAPDH and PPIA in PCCs infected with HSV-1(17^+^)LOX. **C,** mRNA levels of GDNF and NGF in infected. Mean ± SEM, n=3, two-way ANOVA, Holm-Sidak‘s multiple comparison test (* p<0.05, ** p<0.01, *** p<0.001).

**Fig. S2:** FGF-4 is correctly spliced and expressed in HSV-1 infected PCCs. HSV-1 randomly provokes a dysregulation of transcription termination producing non-translated transcripts including introns [79].

**
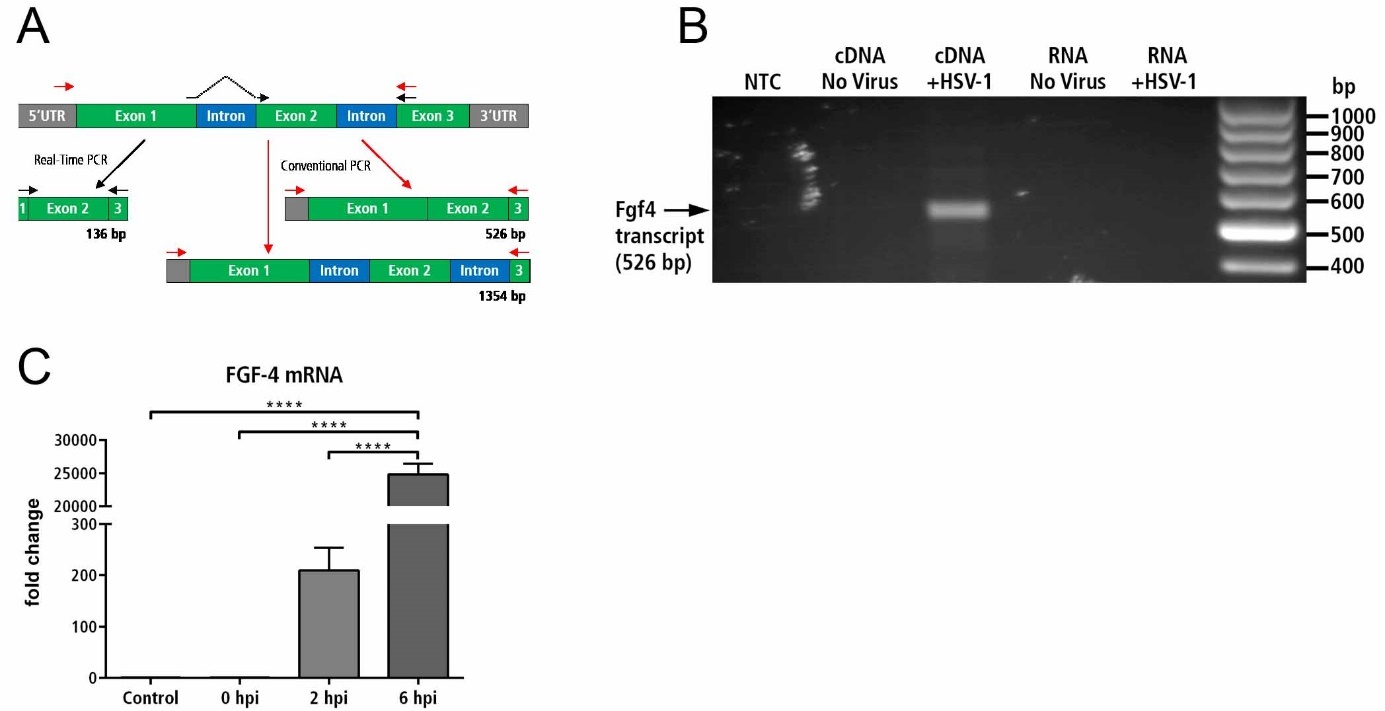
**

**A,** Two primer sets were used for amplification of spliced and non-spliced FGF-4 transcripts in qRT-PCR (black arrows) and conventional RT-PCR (red arrows). **B,** cDNA and RNA from control and HSV-1(17^+^)Lox infected PCCs (MOI 10, 6 hpi) was used with primers able to amplify intron sequences (red arrows in A). NTC = non template control. **C,** Fold change of FGF-4 mRNA in HSV-1(17^+^)Lox infected PCCs (MOI10). Mean ± SEM, one-way ANOVA, Holm-Sidak‘s multiple comparison test (****p> 0.0001).

**Fig S3:** Filtration of HSV-1 conditioned medium removes viral particles.


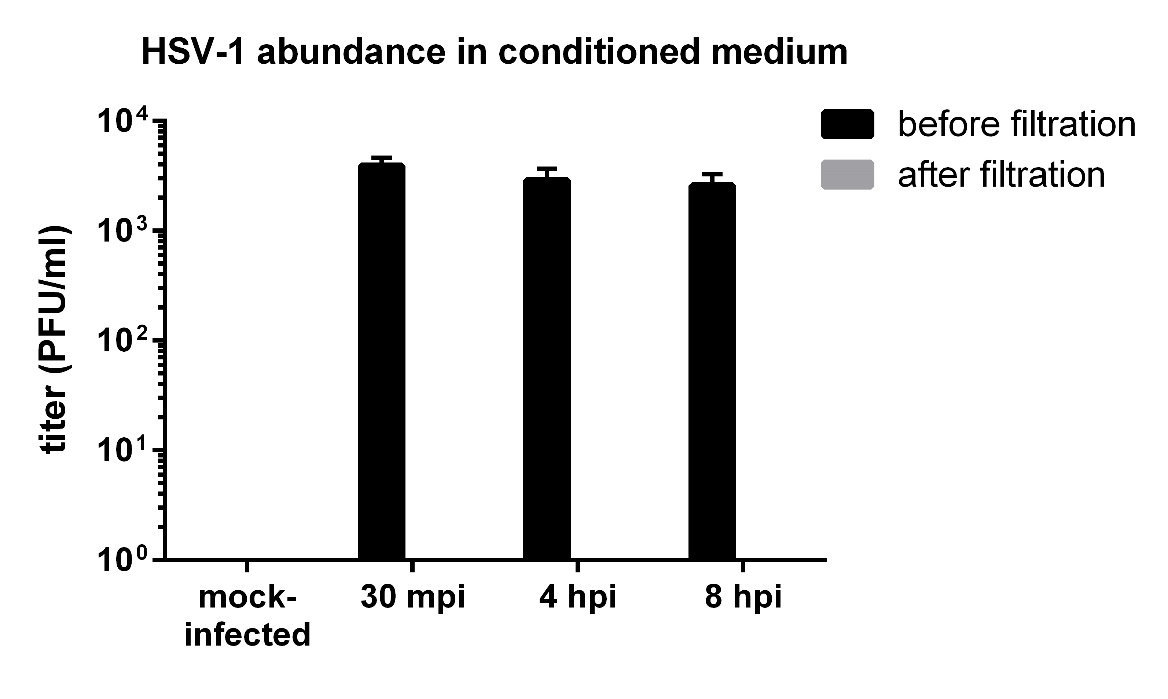


The media of HSV-1 infected cells were filtered followed by plaque assays. We could observe plaques in non-filtered media (detection limit 40 PFU/ml), but no plaques in cells incubated with filtered, HSV-1 conditioned medium. Mean ± SEM, n=5.

**Fig. S4:** Increasing doses of UV-radiation de-activate HSV-1.


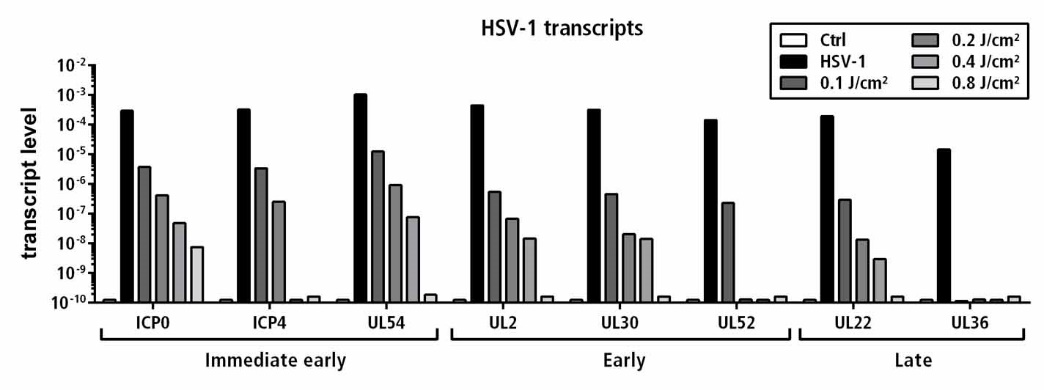


Transcript levels of different viral genes in PCCs infected with untreated or UV-inactivated HSV-1(17^+^)Lox (MOI 10, 4 hpi). Mean from three pooled replicates.

**Fig. S5:** FGF-4 responses are not triggered by MyD88 dependent TLRs or cGAS/STING innate sensing mechanisms.

**
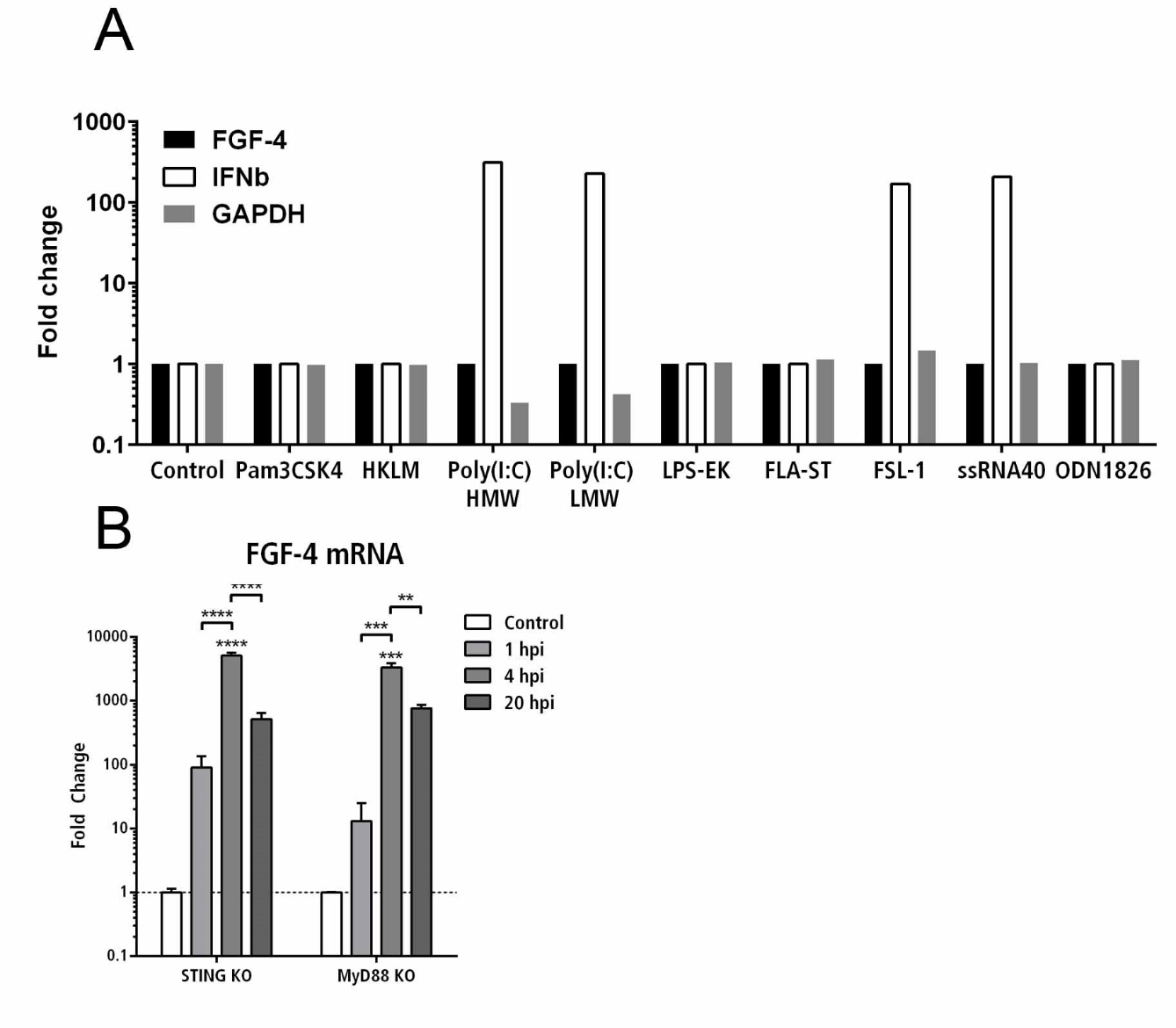
**

**A,** PCCs were incubated with TLR agonists for 6 hours and FGF-4 mRNA levels were quantified. Mean, n=1. **B,** PCCs derived from STING or MyD88 knock-out mice infected with HSV-1 (MOI 10). Mean ± SEM, n=5, one-way ANOVA, Holm-Sidak‘s multiple comparison test (*** p<0.001, ** p<0.01, **** p<0.0001).

**Table S1:** PCR-primer sequences for the detection of murine transcripts or HSV-1 transcripts.

| primer name | sequence |
| --- | --- |
| NH130-Mm-GDNF-F | TGACCAGTGACTCCAATATGCC |
| NH131-Mm-GDNF-R | CCGCTTGTTTATCTGGTGACCT |
| NH134-Mm-NGF-F | CGCATCGAGTGACTTTGGAG |
| NH135-Mm-NGF-R | GGCACAGCATGTTCACTAGGAG |
| NH370-Mm_TNFa_F | TCAGATCATCTTCTCAAAATTC |
| NH371-Mm_TNFa_R | ACTAGTTGGTTGTCTTTGAGAT |
| NH673_Mm_CXCL10_F | TTATTTTTAAAACCGTCCAATA |
| NH674_Mm_CXCL10_R | TTGGGTCTCTTACTACTTTCAA |
| VL_15_FGF4_536_F | CAAGCTCTTCGGTGTGCCTT |
| VL_16_FGF4_651_R | TGAGGGCCATGAACATACCG |
| VL_17_FGF4_435_F | GACACGAGGGACAGTCTTCT |
| VL_18_FGF4_556_R | AAAGGCACACCGAAGAGCTTG |
| NREN_15_Mm_Fgf4_F | ACAGTCTTCTGGAGCTCTCT |
| NREN_16_Mm_Fgf4_R | TACACTCGTCGGTAAAGAAA |
| VL_23_hFGF4_755_F | TATGGCTCGCCCTTCTTCAC |
| VL_24_hFGF4_896_R | CTCGGTTCCCCTTCTTGGTC |
| VL_53_IE_ICP0_F | TCTGCATCCCGTGCATGAAAAC |
| VL_54_IE_ICP0_R | TCACGCCCACTATCAGGTACAC |
| VL_55_IE_ICP4_F | AGGTTGTTGCCGTTTATTGCGT |
| VL_56_IE_ICP4_R | GGGAAGTTGTGGACTGGGAAGG |
| VL_57_E_UL2_F | CCCCGAGTCTTCGGAATGTCTT |
| VL_58_E_UL2_R | TTGACGGTCAGGGTCGTGTTTA |
| VL_59_E_UL30_F | ATTTACAAGGTCCCCCTGGACG |
| VL_60_E_UL30_R | GTTCACCATGCCGTTCACCTTT |
| VL_61_E_UL52_F | GTTGCCTACTTTGACCTGTGCC |
| VL_62_E_UL52_R | TCGTTAGAGAACCGTGGACGAC |
| VL_63_L_UL36_F | TACTCGGCGTAGCCCTTTTGAA |
| VL _64_L_UL36_R | TTTATCGACTACCACGAGGCCG |
| VL_65_L_UL22_F | GACAGCCGAGGCGTAAAATAGC |
| VL_66_L_UL22_R | CCATCTCATCGACGCCCTGTAT |
| NREN_31_Mm_Ptgs2_F | TTGATTGACAGTCCACCTAC |
| NREN_32_Mm_Ptgs2_R | CTCCTTATTTCCCTTCACAC |
| BF_21_18S_F | AGGTAGTGACGAAAAATAACAA |
| BF_22_18S_R | TTAATATACGCTATTGGAGCTG |
